# Stimuli-responsive multifunctional glyconanoparticle platforms for targeted drug delivery and cancer cell imaging†
†Electronic supplementary information (ESI) available: Synthetic procedures and experimental details. See DOI: 10.1039/c6sc05251g



**DOI:** 10.1039/c6sc05251g

**Published:** 2017-03-30

**Authors:** Xumeng Wu, Yu Jia Tan, Hui Ting Toh, Lan Huong Nguyen, Shu Hui Kho, Sing Yian Chew, Ho Sup Yoon, Xue-Wei Liu

**Affiliations:** a Division of Chemistry and Biological Chemistry , School of Physical & Mathematical Sciences , Nanyang Technological University , 21 Nanyang Link , Singapore 637371 , Singapore . Email: xuewei@ntu.edu.sg; b Division of Structural Biology & Biochemistry , School of Biological Sciences , Nanyang Technological University , Singapore 639798 , Singapore; c School of Chemical & Biomedical Engineering , Nanyang Technological University , Singapore 637459 , Singapore; d Lee Kong Chian School of Medicine , Nanyang Technological University , Singapore 308232 , Singapore; e Department of Genetic Engineering , College of Life Sciences , Kyung Hee University , Yongin-si , Gyeonggi-do 446-701 , Republic of Korea

## Abstract

Targeted bioimaging or chemotherapeutic drug delivery to achieve the desired therapeutic effects while minimizing side effects has attracted considerable research attention and remains a clinical challenge. Presented herein is a multi-component delivery system based on carbohydrate-functionalized gold nanoparticles conjugated with a fluorophore or prodrug. The system leverages active targeting based on carbohydrate–lectin interactions and release of the payload by biological thiols. Cell-type specific delivery of the activatable fluorophore was examined by confocal imaging on HepG2 cells, and displays distinct selectivity towards HepG2 cells over HeLa and NIH3T3 cells. The system was further developed into a drug delivery vehicle with camptothecin (CPT) as a model drug. It was demonstrated that the complex exhibits similar cytotoxicity to that of free CPT towards HepG2 cells, and is significantly less cytotoxic to normal HDF and NIH3T3 cells, indicating excellent specificity. The delivery vehicle itself exhibits excellent biocompatibility and offers an attractive strategy for cell-type specific delivery depending on the carbohydrates conjugated in the system.

## Abstract 2

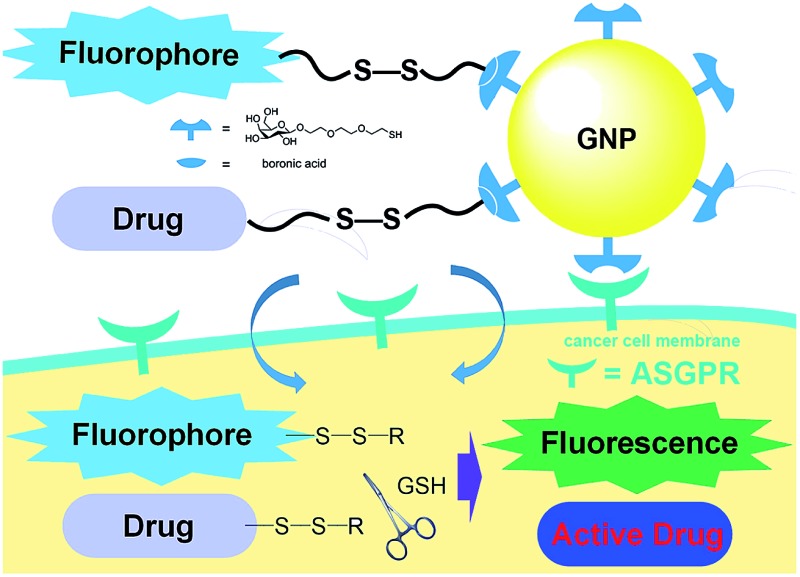
A targeting system with excellent targeting ability is constructed by incorporating carbohydrate-modified gold nanoparticles as vehicles and GSH-responsive species as the payload.

## Introduction

Timely diagnosis and effective treatment of cancer remain challenging due to the lack of early diagnostic technology and the severe side effects associated with chemotherapy resulted from a lack of specificity for cancer cells.^1–9^ Therefore, the development of a targeted system for cancer diagnosis and therapy is highly warranted.^10–12^ Based on the unique cellular characteristics of malignant cells, differentiation between malignant and normal cells is achievable.^13–16^ Hence, targeted fluorescent biomarkers have found great utility in the specific visualization of cancer cells, which enables early-stage detection of cancers instead of relying on advanced morphological changes alone.^17–20^ Moreover, targeted treatment modalities can ideally achieve enhanced drug delivery to specific cancer cells and tissues, thereby dramatically improving the selectivity and efficacy of anti-cancer drugs.^21–24^

Carbohydrates have attracted considerable attention in the development of targeting systems due to their ability to differentiate and recognize cells and the endocytotic uptake resulting from specific carbohydrate–lectin interactions.^25–29^ Among the myriad glycoconjugates available, carbohydrate-functionalized gold nanoparticles (GNPs) are one of the most extensively studied.^30–33^ The colloidal gold provides a globular display of carbohydrates on its surface, mimicking the dense extracellular glycocalyx.^34^ By varying the conjugated carbohydrates and the target lectins expressed on cell surfaces, GNPs have evolved to become valuable tools in the study of carbohydrate–lectin interactions.^35–41^ To this end, our group has previously developed a ‘turn-on/turn-off’ biosensor based on boronic acid-conjugated GNPs, which demonstrates potential for development as a targeted delivery vehicle.^42^

Currently, stimuli-responsive systems, which can be triggered by the unique tumor microenvironment to release their cargo, have also been regarded as one of the most promising strategies to enhance cancer cell selectivity.^10,43^ Considering the much higher concentration of glutathione (GSH) in cancer cells as compared to normal cells, a variety of GSH-responsive systems with cleavable disulfide linkages have been developed for targeted cell imaging and drug delivery.^44–46^

**Scheme 1:**
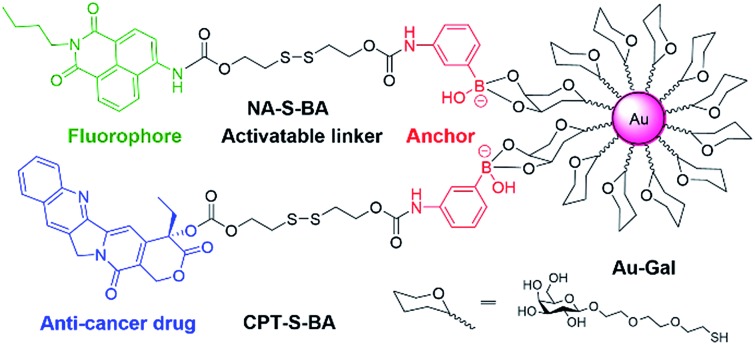
Chemical structures of the activatable fluorophore NA-S-BA and the prodrug CPT-S-BA and the schematic diagram of their conjugation with gold nanoparticles.

In view of these considerations, it is envisioned that a targeted stimuli-responsive system can be established based on the synergistic effect of carbohydrate-functionalized gold nanoparticles as the targeted vehicle and a GSH-responsive disulfide scaffold as the cargo. The payload is linked to the delivery system through reversible cyclic boronate esters that are formed between the carbohydrates and boronic acids. Galactose was first selected as the carbohydrate ligand for the surface modification of gold nanoparticles, to achieve specific recognition of the asialoglycoprotein receptors (ASGPR) expressed on hepatocellular carcinoma cells (HepG2).^47,48^ Thereafter, the boronic acid was conjugated to a naphthalimide fluorophore and camptothecin (CPT, an inhibitor of topoisomerase I for cancer chemotherapy) *via* cleavable disulfide linkers, forming the GSH-activatable fluorophore NA-S-BA and the prodrug CPT-S-BA, respectively (Scheme 1).^49–54^ Enhanced fluorescence, which stems from the GSH-mediated cleavage of NA-S-BA, was observed in ASGPR-expressing HepG2 cells as compared to ASGPR-deficient cancer cells (HeLa cells) and ASGPR-deficient non-tumorigenic cells (NIH3T3 cells).^55,56^ Accordingly, nanoparticles conjugated with CPT-S-BA exhibit distinct selectivity for cancer cells over normal cells, without compromising the chemotherapeutic efficacy of the native CPT. A noteworthy advantage is that by simply changing the carbohydrate moiety on the GNP, this system possesses the potential to target a variety of cancer cells based on the lectins expressed on the cell surface and the corresponding conjugated carbohydrates. To the best of our knowledge, this is the first combination of carbohydrate-modified gold nanoparticles with activatable disulfide linkers and reversible boronate esters incorporated into the delivery system.

## Results and discussion

The synthetic routes to NA-S-BA, NA-C-BA, CPT-S-BA and the functionalized gold nanoparticles are depicted in ESI,† Experimental section. The key intermediate compound NA-NH_2_ was synthesized according to established procedures with minor modifications.^57^ In brief, the disulfide linker 2,2′-dithiodiethanol was conjugated with NA-NH_2_ and CPT to form NA-S and CPT-S in a reaction mediated by triphosgene. Subsequently, the boronic acid unit was introduced by conjugating 3-aminophenylboronic acid pinacol ester with NA-S or CPT-S in the presence of triphosgene and DMAP. The desired NA-S-BA and CPT-S-BA were obtained after a mild deprotection of the pinacol functionality in the boronic acid unit. Meanwhile, the control compound NA-C-BA, with an uncleavable linker (“C–C”), was also synthesized using the same method as for NA-S-BA. Thiol-modified β-galactoside was synthesized and conjugated onto citrate-gold nanoparticles with a diameter of approximate 15 nm (ESI, Fig. S1†), forming stable Au-Gal nanoparticles. The boronic acid-conjugated payload was further incubated with Au-Gal to form Au-Gal-BA (galactose-modified gold nanoparticles conjugated with NA-S-BA) and Au-Gal-BA(CPT) (galactose-modified gold nanoparticles conjugated with CPT-S-BA).

**Fig. 1:**
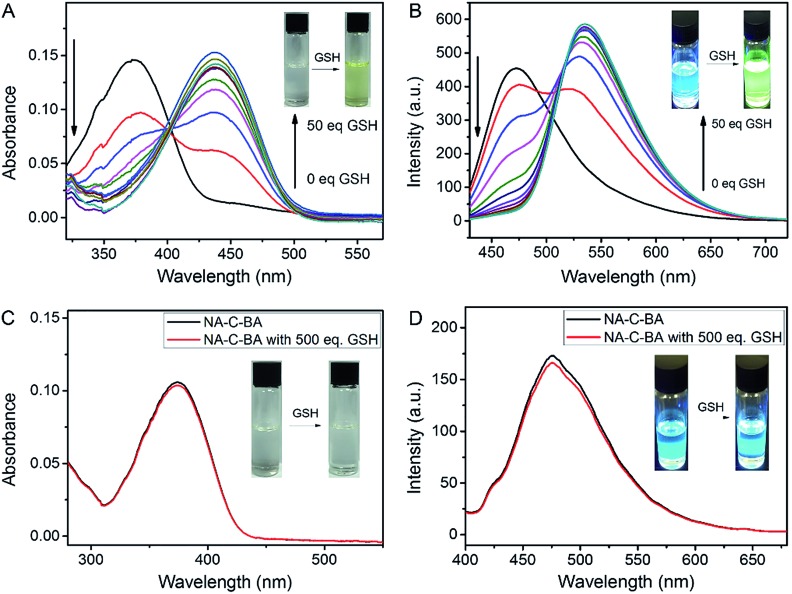
Absorption and emission changes of (A, B) NA-S-BA (10 µM) and (C, D) control NA-C-BA (10 µM) in the presence of GSH in DMSO/PBS solution (1 : 1, v/v, pH = 7.4, 10 mM). Insets (A) and (C): color changes observed in NA-S-BA and NA-C-BA solutions upon addition of GSH. Insets (B) and (D): visible fluorescence changes in NA-S-BA and NA-C-BA upon addition of GSH. Each point was recorded after exposure to GSH for 1 h at 37 °C, *λ*_ex_ = 405 nm. Note: here the isosbestic point of 405 nm is chosen as the excitation wavelength for an accurate comparison of the fluorescence intensity before and after GSH-induced cleavage of the disulfide bond.

**Fig. 2:**
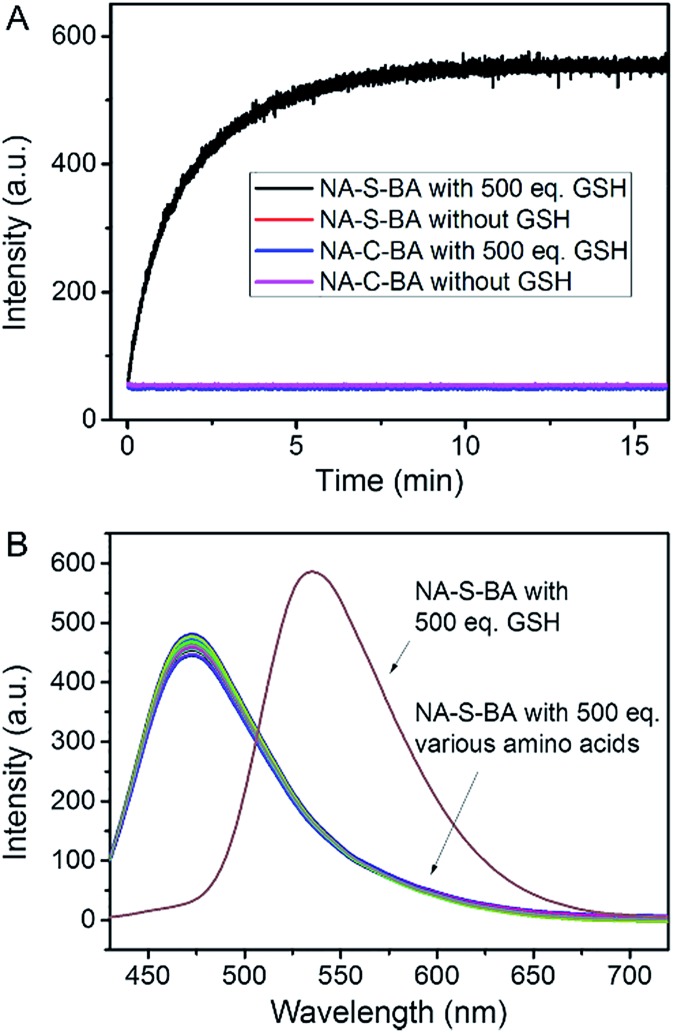
(A) Changes in fluorescence intensity at 535 nm for NA-S-BA and NA-C-BA (10 µM) in DMSO/PBS solution (1 : 1, v/v, pH = 7.4, 10 mM) in the presence (black and blue) and absence (red and purple) of GSH (500 eq.) over time, *λ*_ex_ = 405 nm. Data was recorded every 0.5 s. (B) Fluorescence response of NA-S-BA (10 µM) upon addition of various amino acids including Ala, Leu, Ile, Val, Pro, Phe, Met, Trp, Gly, Ser, Gln, Thr, Asn, Tyr, Asp, Glu, Lys, Arg, and His (500 eq.). Each spectrum was recorded after exposure to GSH for 1 h at 37 °C, *λ*_ex_ = 405 nm.

The spectroscopic properties of the activatable fluorophore NA-S-BA were first tested to investigate the efficiency of the GSH-mediated cleavage of the disulfide linker. Due to the typical donor–π bridge–acceptor (D–π–A) structure,^58,59^ NA-S-BA exhibits broad absorption and fluorescence peaks centered at 374 nm and 472 nm, respectively, and appears as a pale-yellow solution with blue fluorescence (Fig. 1A). On the other hand, under the same conditions, the key intermediate NA-NH_2_ gives distinctly different spectra with absorption at 437 nm and strong green fluorescence at 535 nm (ESI, Fig. S2†). Subsequently, the responsiveness to GSH was investigated *via* the change in the spectral properties of NA-S-BA upon addition of GSH. As shown in Fig. 1, after the reaction with GSH at 37 °C, a red-shift of 63 nm was observed simultaneously in the absorption and fluorescence spectra of NA-S-BA, with concurrent changes in the color of the solution (from almost colorless to yellow) and its fluorescence when irradiated with light (blue to green). Moreover, the isosbestic point in the absorption spectra at around 405 nm indicates the generation of a new compound after interaction with GSH. The apparent differences between NA-S-BA and NA-NH_2_ can be ascribed to the carbamate structure in the former, which masks the electron-donating ability of the nitrogen atom. Cleavage of the disulfide bond reveals the amino group, restoring the strong intramolecular charge transfer (ICT) process. Meanwhile, control NA-C-BA, which comprises stable C–C bonds instead of a disulfide linker, reveals a negligible change in photophysical properties even in the presence of excess GSH (Fig. 1C and D). Otherwise, the time-dependent fluorescence spectra of NA-S-BA in the absence of GSH and NA-C-BA with or without GSH addition display similar results (Fig. 2A). The results clearly demonstrate that the fluorescence change can only be induced by the simultaneous existence of NA-S-BA and GSH, hence the disulfide bond is integral to the GSH-induced fluorescence release.

**Scheme 2:**
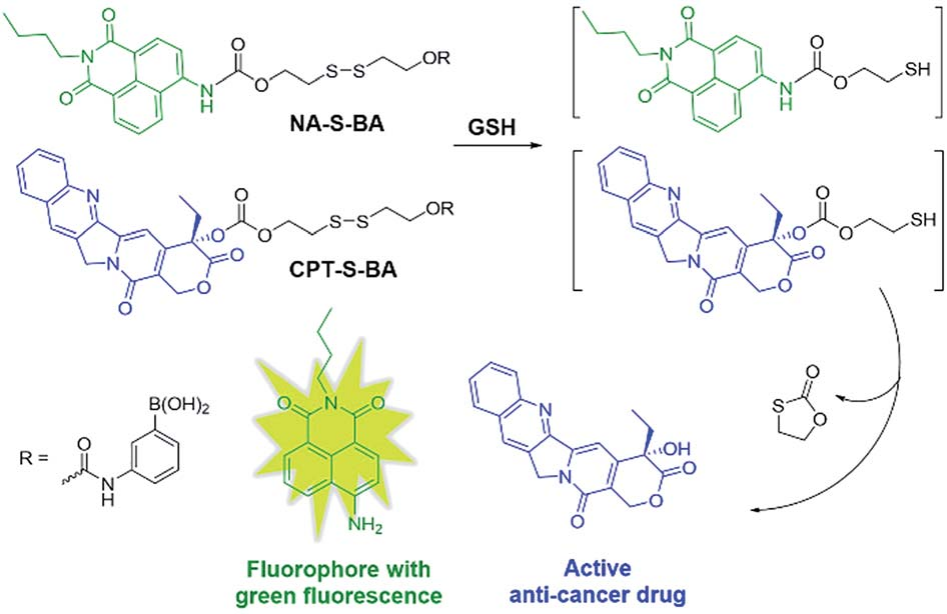
Proposed fluorescence and CPT release mechanism by treatment with GSH.

The spectroscopic data of the GSH-treated NA-S-BA was further compared with that of NA-NH_2_, which is hypothesized to be the final product upon interaction with GSH. As illustrated in ESI, Fig. S3,† the perfectly identical positions and shapes of the absorption and fluorescence peaks indicate that after the reaction with GSH, NA-NH_2_ is generated as the product and accounts for the fluorescence source. Combining the spectroscopic results and the MS analysis results indicating the formation of NA-NH_2_ and free CPT upon interaction with GSH (ESI, Fig. S4†), it can be confirmed that after cleavage of the disulfide bond by thiol-containing GSH, tandem intramolecular cyclization occurs as shown in Scheme 2.^60^ Notably, the extremely large Stokes shift of 98 nm of NA-NH_2_, which results from the intramolecular charge transfer (ICT) from the amino unit (donor) to the naphthalimide unit (acceptor), is desirable for high quality optical imaging due to the enhancement in signal fidelity.^19,61,62^

The feasibility of applying this model in biological systems was evaluated by examining the influence of other biomolecules, such as amino acids. As shown in Fig. 2B and ESI, Fig. S5,† no appreciable change in the fluorescence and absorption spectra of NA-S-BA could be observed when it was treated with thiol-free amino acids. On the other hand, similar results to treatment with GSH could be obtained in the presence of 1,4-dithiothreitol (DTT), cysteine (Cys), and homocysteine (Hcy), owing to their thiol-containing structures (ESI, Fig. S6†). However, the potential interference of Cys and Hcy could be neglected due to their comparatively low concentrations in contrast to the high concentration of GSH in the cytoplasm (1–15 mM).^63–66^ The effect of pH variation on the GSH-induced fluorescence changes of NA-S-BA was also investigated. As shown in ESI, Fig. S7,† NA-S-BA remains stable and non-fluorescent within a pH range of 3.5–9, and produces the aforementioned activatable fluorescence response to GSH across the pH range of 5 to 9. Hence, GSH-induced disulfide bond cleavage and the subsequent fluorescence release can be achieved under physiological conditions without potential biological interference.

**Fig. 3:**
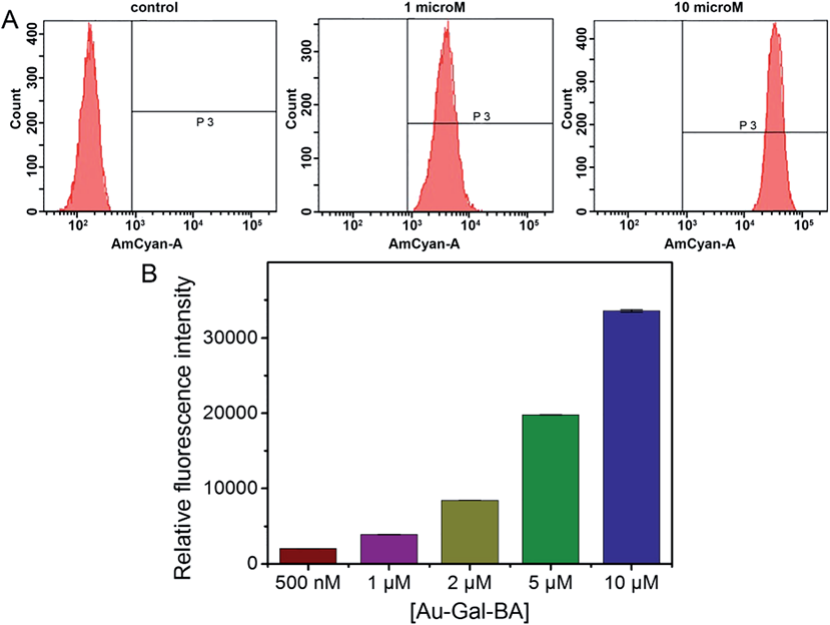
Concentration-dependent uptake of Au-Gal-BA in HepG2 cells as determined by flow cytometry. (A) Histograms of HepG2 cells with different concentrations of Au-Gal-BA. (B) Relative fluorescence intensities expressed with respect to control cells as mean ± SD (*n* = 3). Measured using flow cytometry (AmCyan channel, BD 525/50 filter).

Having established the favorable spectroscopic properties of NA-S-BA and CPT-S-BA, cellular studies were conducted to assess the potential applicability of the stimuli-responsive system as a bioimaging and drug delivery model. To confirm the role of carbohydrate–lectin binding in the targeting ability of the complex to the desired cell-type, HepG2 was first selected for the study as the overexpression of asialoglycoprotein receptors (ASGPR) on hepatic cells is well-established.^48^ The cellular uptake of Au-Gal-BA was examined by incubating HepG2 cells with increasing concentrations of Au-Gal-BA and determined by flow cytometry (Fig. 3). It is evident that the uptake is concentration-dependent, with the fluorescence intensity increasing proportionately with the amount of Au-Gal-BA added.

**Fig. 4:**
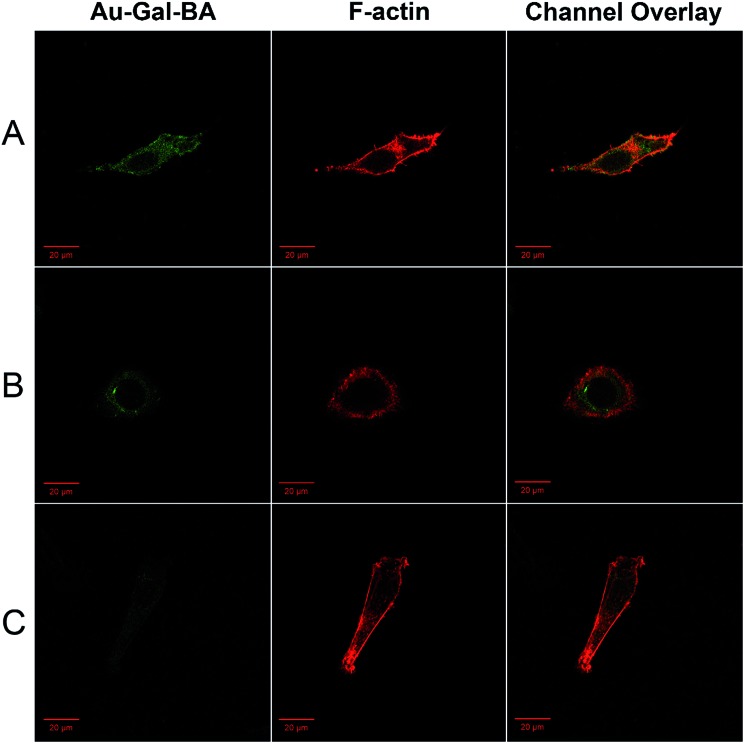
Confocal microscopy images of (A) HepG2, (B) HeLa and (C) NIH3T3 cells incubated with Au-Gal-BA. Cells were treated with the complexes for 2 h and the cytoskeletons were stained with Alexa Fluor 633 phalloidin. Cell images were acquired using excitation wavelengths of 488 nm and 633 nm, and emission filters in the ranges of 501–602 nm and 638–747 nm for the imaging of Au-Gal-BA (green) and phalloidin (red), respectively. The last panel shows the overlay of both channels.

To determine the cell-type specificity of the Gal-targeting ligands on the Au-Gal-BA complexes, cellular uptake in ASGPR-overexpressing HepG2 was compared with that in HeLa and NIH3T3 cells. Earlier studies showed that cervical carcinoma HeLa cells and mouse fibroblast NIH3T3 cells have negligible ASGPR expression. As is evident in Fig. 4A, the fluorescence intensity corresponding to uptake and cleavage of Au-Gal-BA was highest in HepG2, due to the overexpression of ASGPR and high intracellular GSH levels.^67^ A discernable difference in fluorescence intensity is observed in HeLa cells (Fig. 4B), which, despite having lower ASGPR expression, are also capable of disulfide-cleavage due to the presence of high GSH levels.^68^ The contrast is most significant in NIH3T3 cells, which express neither ASGPR nor high levels of GSH (Fig. 4C).^69^ It is apparent that the fluorescence signal originating from the cleavage product of Au-Gal-BA is weakest in NIH3T3. This is primarily due to the fact that the absence of ASGPR results in low cellular uptake, and cleavage of NA-S-BA to form fluorescent NA-NH_2_ is hampered by the low intracellular GSH concentration. Besides cell-type selectivity, another important parameter that determines the practical utility of a bioimaging system is the inherent cytotoxicity. As is evident in ESI, Fig. S8,† Au-Gal-BA is non-toxic to all three cell types across the range of concentrations tested. Thus, the fluorescent payload in Au-Gal-BA can be taken up efficiently by the target cells, yet is well-tolerated and exhibits excellent biocompatibility.

In order to determine the intracellular localization fate of the fluorescent payload upon cellular uptake, imaging experiments were conducted with lysosome-, mitochondria- and endoplasmic reticulum (ER)-specific staining reagents. As is evident in ESI, Fig. S9,† no co-localization was observed with the Lyso- or Mito-tracker. Diffused fluorescence of the payload can be seen within the cytosolic environment of the cells, indicating the ability of the compound to escape from the lysosomes, a key consideration in the delivery of anti-cancer drugs. On the contrary, the fluorescence co-localized well with the ER-tracker, with overlapping signals from the red fluorescence of the ER-tracker and the green fluorescence in the Au-Gal-BA channel. This observation is postulated to be due to the cleavage of the S–S bond in the ER, resulting in the release of the fluorescent payload in the ER-compartment (ESI, Fig. S9C†).^46^

**Fig. 5:**
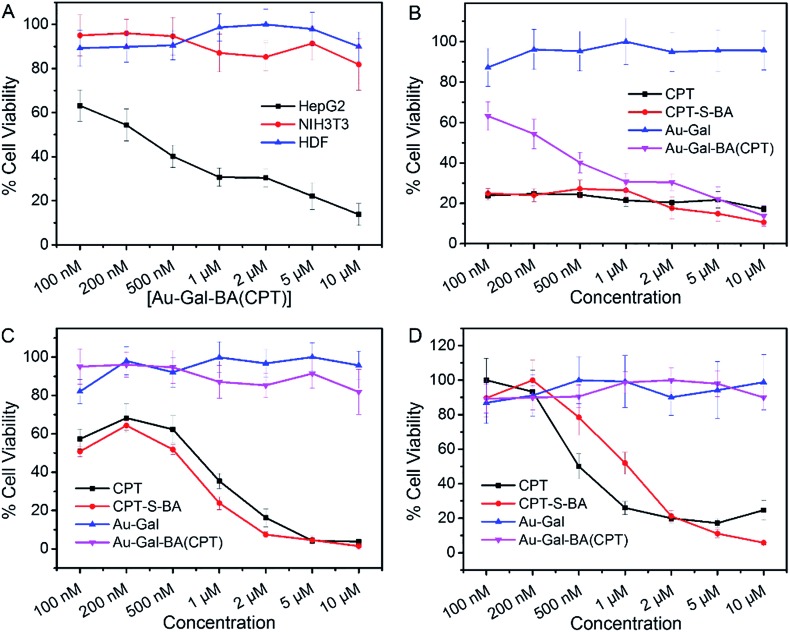
(A) Comparative viability of HepG2, NIH3T3 and HDF cells incubated with increasing concentrations of the Au-Gal-BA(CPT) complex for 72 h. Comparison of cell viability in (B) HepG2, (C) NIH3T3 and (D) HDF cells with increasing concentrations of CPT, CPT-S-BA, Au-Gal and Au-Gal-BA(CPT). Measured using WST-1 assay, with absorbance quantified at 450 nm (reference: 650 nm). Data is represented as mean ± SEM (*n* ≥ 3).

The potential applicability of the Au-Gal-BA model as a targeted drug delivery system was further investigated by conjugating a chemotherapeutic prodrug, CPT-S-BA, to the delivery vehicle, forming the Au-Gal-BA(CPT) complex. Spectroscopic analysis of Au-Gal-BA(CPT) confirmed the successful conjugation of the drug onto the Au-Gal nanoparticles (ESI, Fig. S10†). When HepG2 cells were incubated with increasing concentrations of Au-Gal-BA(CPT), a significant decrease in cell viability was observed (Fig. 5A). This decrease is noted to be dose-dependent, with less than 15% of the cells viable after incubation with 10 µM Au-Gal-BA(CPT). On the contrary, when NIH3T3 cells, which do not express ASGPR, were incubated with the complexes, no notable cytotoxicity was observed. Even when the concentration was increased to the micromolar range, more than 80% of the NIH3T3 cells remained viable. Human Dermal Fibroblasts (HDFs), model primary human adult somatic cells, were further incubated with Au-Gal-BA(CPT) as a control to investigate the effect of the prodrug on normal human cells (Fig. 5A). Negligible cytotoxicity was observed even at high concentrations, which is consistent with the experimental hypothesis that the vehicle is capable of drug delivery to specific cancer cells with minimal cytotoxicity to normal cells.

It is postulated that in ASGPR-expressing HepG2 cells, recognition and binding of the galactose-appended Au-Gal nanoparticles to ASGPR results in the concomitant release of the CPT-S-BA payload. The boronic acid moiety acts as a delivery agent which allows the prodrug to be delivered across the cell membrane.^70,71^ Upon cellular entry, the high concentration of GSH in the cytoplasmic environment of HepG2 cancer cells leads to the cleavage of the disulfide linkage in the ER, thus releasing the CPT chemotherapeutic drug. Thereafter, CPT diffuses into the nucleus, where it is able to bind to DNA topoisomerase I and inhibit DNA replication, leading to cell death.^72^ In ASGPR-deficient NIH3T3 and HDF cells, the lack of galactose-binding receptors results in an inability to take up Au-Gal-BA(CPT) efficiently. Moreover, as normal cells express much lower concentrations of GSH than malignant cells, the cleavage of the S–S bond in CPT-S-BA is expected to be slower in NIH3T3 and HDF cells as compared to HepG2. Hence, the overall uptake of Au-Gal-BA(CPT) and intracellular release of CPT are considerably less efficient in the normal NIH3T3 and HDF cells, leading to much lower observed cytotoxicity.

In order to investigate the biocompatibility of the Au-Gal delivery vehicle, the cells were also incubated with increasing concentrations of Au-Gal (Fig. 5). As is evident in the results presented, the model demonstrates excellent biocompatibility and no cytotoxicity was observed towards all three cell types tested. The cytotoxicity profiles of CPT-S-BA and CPT are also identical in HepG2, NIH3T3 and HDF (Fig. 5B–D). This indicates that while the boronic acid component alone is capable of intracellular entry, the toxicity is ascribable to the chemotherapeutic effect of CPT and does not arise from the boronic acid functionality. This is congruent with the reported biocompatibility of boronic acid with human physiology.^73,74^

**Fig. 6:**
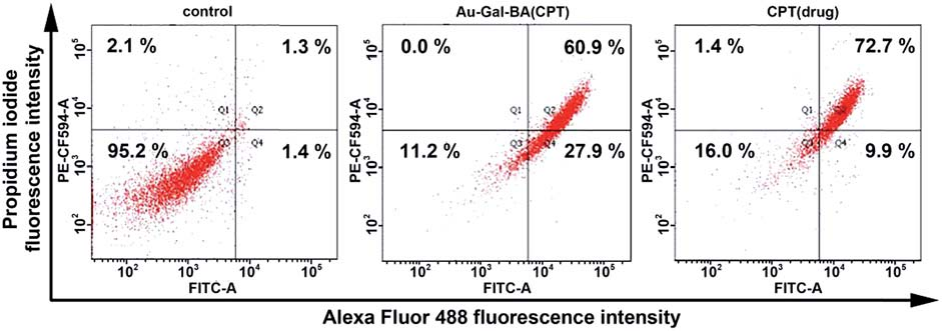
Annexin V/PI assay of HepG2 control, HepG2 incubated with Au-Gal-BA(CPT), and CPT. Fluorescence was analyzed *via* flow cytometry (PE-CF594 and FITC channel). Inserted numbers indicate percentage of cells in each area.

Finally, an Annexin V/PI assay was used to quantify the percentages of live and apoptotic cells when incubated with Au-Gal-BA(CPT) (Fig. 6). In the absence of any chemotherapeutic agent, most of the cells are viable. As anticipated, the addition of the Au-Gal-BA(CPT) complex was able to induce apoptosis in HepG2 cells, and the total apoptotic cell population increased significantly to 88.8%, a drastic increase as compared to the control cells. Cells treated with CPT were analyzed as a reference, and it is evident that the results obtained with Au-Gal-BA(CPT) are comparable with those obtained using CPT, with the total apoptotic population accounting for 82.6% of the cell population in the latter condition.

## Conclusions

In summary, we have developed a stimuli-responsive model for both bioimaging and delivery of chemotherapeutic drugs to target cancer cells. The carbohydrates coated on the GNP act as targeting ligands by binding to cell surface lectins, concomitantly releasing the boronic acid-linked payload, which is internalized into the cells. High intracellular GSH levels then result in disulfide bond cleavage, which triggers an intramolecular cyclization that leads to the release of the fluorophore and a red-shifted fluorescence enhancement. The uptake of Au-Gal-BA was further studied through flow cytometry and confocal microscopy, and demonstrates selectivity for target HepG2 cells. When CPT was incorporated as a model prodrug, selective targeting of HepG2 cells over the NIH3T3 control and normal HDF cells was achieved, with significant cytotoxicity observed only towards the target HepG2 cells. The delivery vehicle itself is non-toxic and biocompatible, indicating the potential to develop it into a useful bioimaging tool, as well as a targeted drug delivery system in translational research. The most prominent advantage of this system is the ability to target different cells based on the extracellular lectins expressed on the cell surface and the corresponding carbohydrates coated on the GNP.

## Experimental section

### Materials and characterizations

All reagents and solvents were purchased from commercial sources and were of analytical grade. ^1^H and ^13^C NMR spectra in CDCl_3_ or DMSO-*d*_6_ were recorded on a Bruker AV 300 MHz NMR instrument with tetramethylsilane (TMS) as the internal standard. Data for ^1^H NMR spectra is reported as follows: chemical shift (ppm) and multiplicity (s = singlet, d = doublet, t = triplet, q = quartet, m = multiplet). Data for ^13^C NMR spectra is reported in ppm. High Resolution Mass Spectroscopy (HRMS) spectra were recorded on a Waters Q-Tof Premier™ Mass Spectrometer. UV/Vis spectra were measured with a Varian Cary 100 spectrophotometer (1 cm quartz cell). Emission spectra were measured with a Varian Cary Eclipse (1 cm quartz cell). The time dependent fluorescence study was conducted by fluorescence induction *in situ* and measured with a Varian Cary Eclipse (1 cm quartz cell) at 37 °C. Purification by flash column chromatography was carried out using silica gel 60 (0.010–0.063 mm) with eluents as noted in the experimental data sections for the respective compounds. Deionized water was used in the preparation of all samples.

### Synthesis of NA-S-BA

A mixture of NA-S-BAP (30 mg, 0.04 mmol), sodium periodate (46 mg, 0.22 mmol) and ammonia acetate (17 mg, 0.22 mmol) in acetone/water (1 : 1, v/v, 10 mL) was stirred overnight at room temperature. After the removal of acetone, the precipitate was collected and washed with hexane to afford a quantitative yield of NA-S-BA as a pale yellow solid (24 mg). Melting point: 137–140 °C. ^1^H NMR (300 MHz, DMSO-*d*_6_, ppm): *δ* 9.348 (s, 1H, NH), 8.735 (d, *J* = 8.7 Hz, 1H, Ph-H), 8.618 (m, 3H, Ph-H), 8.342 (d, *J* = 8.4 Hz, 1H, Ph-H), 7.951 (s, 1H, Ph-H), 7.838 (t, *J* = 7.8 Hz, 1H, Ph-H), 7.620 (d, *J* = 6.9 Hz, 1H, Ph-H), 7.530 (d, *J* = 7.5 Hz, 1H, Ph-H), 7.248 (t, *J* = 7.8 Hz, 1H, Ph-H), 7.125 (s, 1H, NH), 4.558 (t, *J* = 6.3 Hz, 2H, –O–CH_2_), 4.440 (t, *J* = 6.3 Hz, 2H, OH–CH_2_), 4.141 (t, *J* = 7.5 Hz, 2H, N–CH_2_), 3.050–3.200 (m, 4H, –CH_2_–), 1.709 (m, 2H, N–CH_2_–CH_2_), 1.474 (m, 2H, –CH_2_–CH_3_), 0.984 (t, 3H, CH_3_). ^13^C NMR (75 MHz, DMSO-*d*_6_, ppm): *δ* 163.932, 163.386, 154.355, 153.810, 141.042, 138.544, 132.072, 131.370, 129.815, 128.867, 128.791, 128.081, 126.877, 124.527, 122.704, 119.111, 117.735, 63.357, 62.404, 37.314, 37.309, 30.141, 20.264, 14.185. Mass spectrometry (ESI-MS, *m*/*z*): [M + H]^+^ calcd for C_28_H_31_BN_3_O_8_S_2_, 612.1646; found, 612.1654.

### Synthesis of NA-C-BA

The compound NA-C-BA was synthesized using the same procedure as in the synthesis of NA-S-BA. NA-C-BA was afforded as a brown solid (22 mg): yield 98%. Melting point: 125–128 °C. ^1^H NMR (300 MHz, DMSO-*d*_6_, ppm): *δ* 10.237 (s, 1H, NH), 9.466 (s, 1H), 8.703 (d, *J* = 8.4 Hz, 1H, Ph-H), 8.503 (q, 2H, Ph-H), 8.175 (d, *J* = 8.4 Hz, 1H, Ph-H), 7.947 (s, 2H), 7.740–7.890 (m, 2H, Ph-H), 7.520 (d, *J* = 7.8 Hz, 1H, Ph-H), 7.434 (d, *J* = 7.2 Hz, 1H, Ph-H), 7.229 (t, *J* = 7.8 Hz, 1H, Ph-H), 4.219 (t, *J* = 6.3 Hz, 2H, CH_2_), 4.072 (m, 4H, CH_2_), 1.600–1.740 (m, 6H, CH_2_), 1.250–1.500 (m, 6H, CH_2_), 0.933 (t, 3H, CH_3_). ^13^C NMR (75 MHz, DMSO-*d*_6_, ppm): *δ* 163.946, 163.393, 154.623, 154.208, 141.312, 138.774, 132.151, 131.354, 129.802, 128.804, 128.679, 128.066, 126.791, 124.325, 122.689, 118.632, 117.437, 65.493, 64.404, 30.142, 28.984, 28.863, 25.547, 20.261, 14.179. Mass spectrometry (ESI-MS, *m*/*z*): [M + H]^+^ calcd for C_30_H_35_BN_3_O_8_, 576.2517; found, 576.2505.

### Synthesis of CPT-S-BA

The compound CPT-S-BA was synthesized using the same procedure as in the synthesis of NA-S-BA, utilizing THF/water (4 : 1, v/v) as the solvent. CPT-S-BA was afforded as a white solid (16 mg): yield 80%. Melting point: 225–227 °C. ^1^H NMR (300 MHz, DMSO-*d*_6_, ppm): *δ* 9.539 (s, 1H, NH), 8.683 (s, 1H, Ph-H), 8.133 (q, 2H, Ph-H), 7.800–8.080 (m, 3H, Ph-H & B-OH), 7.734 (m, 2H, Ph-H), 7.469 (m, 2H, Ph-H), 7.221 (t, *J* = 7.8 Hz, 1H, Ph-H), 7.101 (s, 1H, Ph-H), 5.527 (s, 2H, CH_2_), 5.315 (s, 2H, CH_2_), 4.353 (t, *J* = 6.0 Hz, 2H, CH_2_), 4.249 (t, *J* = 6.0 Hz, 2H, CH_2_), 3.016 (m, 4H, CH_2_), 2.175 (m, 2H, CH_2_), 0.920 (t, 3H, CH_3_). ^13^C NMR (75 MHz, DMSO-*d*_6_, ppm): *δ* 167.513, 156.964, 153.711, 153.264, 152.700, 148.363, 146.744, 145.191, 138.547, 132.086, 130.914, 130.269, 129.456, 129.002, 128.840, 128.489, 128.218, 128.079, 119.647, 94.842, 78.374, 66.938, 66.748, 62.272, 50.814, 37.263, 36.708, 30.786, 8.010. Mass spectrometry (ESI-MS, *m*/*z*): [M + H]^+^ calcd for C_32_H_31_BN_3_O_10_S_2_, 692.1544; found, 692.1571.
