# Supplementary material for: Stimuli-responsive multifunctional glyconanoparticle platforms for targeted drug delivery and cancer cell imaging
Source: Chem Sci. 2017 Mar 30;8(5):3980–8. doi: 10.1039/c6sc05251g (PMC5433505; doi:10.1039/c6sc05251g)
Supplement: Supplementary file 1 [file SC-008-C6SC05251G-s001.pdf]

Electronic Supplementary Material (ESI) for Chemical Science.
This journal is © The Royal Society of Chemistry 2017

Stimuli-responsive Multifunctional Glyconanoparticle Platforms for Targeted Drug Delivery and Cancer Cell Imaging

Xumeng Wu,[‡a] Yu Jia Tan,[‡a] Hui Ting Toh,[b] Lan Huong Nguyen,[c] Shu Hui Kho,[a] Sing Yian Chew,[c,d] Ho Sup Yoon[b,e] and Xue-Wei Liu[*a]

[a] Division of Chemistry and Biological Chemistry, School of Physical & Mathematical Sciences, Nanyang Technological University, 21 Nanyang Link, Singapore 637371, Singapore. E-mail: xuewei@ntu.edu.sg

[b] Division of Structural Biology & Biochemistry, School of Biological Sciences, Nanyang Technological University, Singapore 639798, Singapore.

[c] School of Chemical & Biomedical Engineering, Nanyang Technological University, Singapore 637459, Singapore.

[d] Lee Kong Chian School of Medicine, Nanyang Technological University, Singapore 308232, Singapore.

[e] Department of Genetic Engineering, College of Life Sciences, Kyung Hee University, Yongin-si, Gyeonggi-do 446701, Republic of Korea.

Experimental Section

The intermediate compound $NA-NH_2$ was synthesized according to established procedures.[S1]

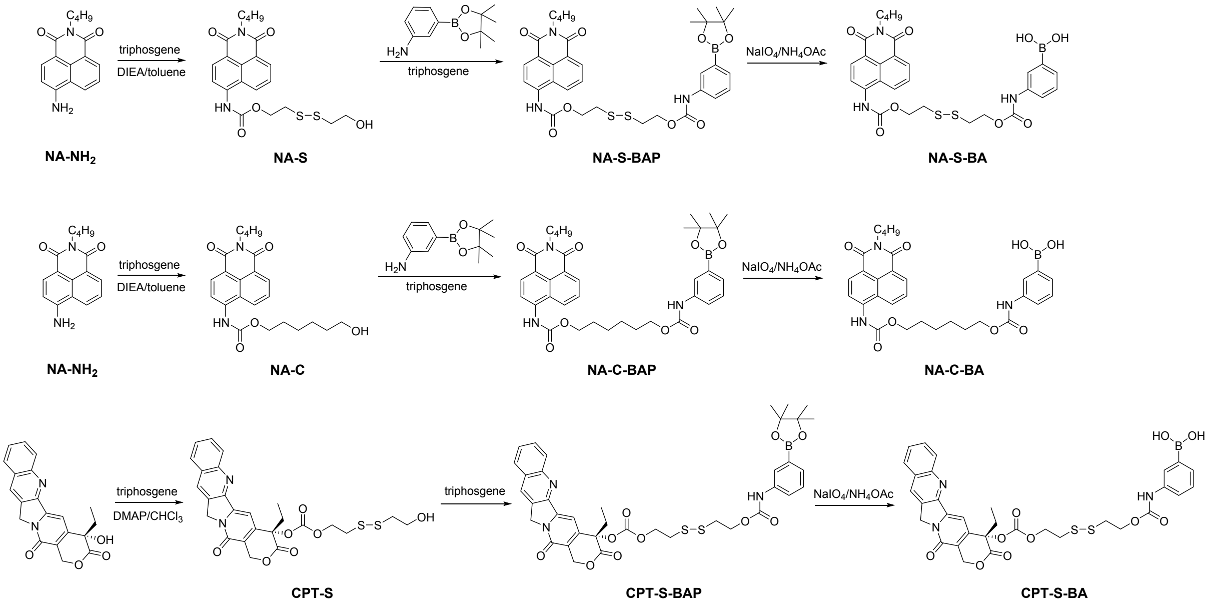

Scheme S1. Synthetic route of NA-S-BA, NA-C-BA and CPT-S-BA.

***Synthesis of NA-S*:** To a mixture of $NA-NH_2$ (240 mg, 0.88 mmol), triphosgene (296 mg, 1 mmol) and dry toluene (20 mL) was added DIEA (412 mg, 3.2 mmol) dropwise under an argon atmosphere at room temperature. The resulting solution was refluxed under argon protection for 3 h. After removal of unreacted phosgene gas by flushing with argon gas, a solution of 2,2'-dithiodiethanol (904 mg, 90%, 5.28 mmol) in CH_2Cl_2/THF (1:1, 10 mL) was added to the mixture and the reaction mixture was stirred overnight at room temperature. After removing the solvent under reduced pressure, the crude product was purified by silica gel chromatography using ethyl acetate/PE (v/v, 1:1) as the eluent to afford Na-S as a yellow solid (160 mg): Yield 40%. Melting point: 142-145 °C. ^{1}H NMR (300 MHz, $CDCl_3$, ppm): δ 8.628 (t, J = 8.7 Hz, 1H, Ph-H), 8.380 (d, J = 8.4 Hz, 1H, Ph-H), 8.247 (d, J = 8.7 Hz, 1H, Ph-H), 7.801(t, J = 8.1 Hz, 1H, Ph-H), 7.691 (s, 1H, Ph-H), 4.857 (t, J = 6.0 Hz, 2H, N-CH_2), 4.198 (t, J = 7.5 Hz, 2H, -O-CH_2), 3.980 (d, J = 8.7 Hz, 2H, OH-CH_2), 3.096 (t, J = 6.0 Hz, 2H, -CH_2), 2.983 (t, J = 5.7 Hz, 2H, -CH_2), 2.214 (s, 1H, OH), 1.742 (q, 2H, N-CH_2-CH_2-), 1.484 (q, 2H, CH_3-CH_2), 1.001 (t, 3H, -CH_3). ^{13}C NMR (75 MHz, $CDCl_3$, ppm): δ 164.102, 163.628, 153.028, 138.825, 132.371,

131.222, 128.868, 126.617, 126.116, 123.386, 123.098, 118.038, 117.034, 63.847, 60.577, 41.566, 40.240, 37.520, 30.195, 20.376, 13.831. Mass spectrometry (ESI-MS, m/z): $[M + H]^+$ calcd for $C_{21}H_{25}N_2O_5S_2$, 449.1205; found, 449.1194.

***Synthesis of NA-S-BAP*:** A mixture of triphosgene (96 mg, 0.32 mmol), DMAP (336 mg, 2.75 mmol) and 4-aminophenylboronic acid pinacol ester (200 mg, 0.92 mmol) in 10 mL of anhydrous chloroform was stirred under argon protection for 0.5 h at room temperature. Then a solution of NA-S (330 mg, 0.74 mmol) in anhydrous chloroform was added. The resulting mixture was stirred overnight at room temperature. After the removal of the solvent under reduced pressure, the crude product was purified by flash silica gel chromatography using ethyl acetate/dichloromethane (v/v, 1:6) as the eluent to afford NA-S-BAP as a pale yellow solid (300 mg): Yield 58.6%. Melting point: 100-105 °C. ^{1}H NMR (300 MHz, $CDCl_3$, ppm): δ 8.611 (q, 2H, Ph-H), 8.318 (t, 2H, Ph-H), 7.975 (s, 1H, NH), 7.654 (m, 2H, Ph-H), 7.548 (d, J = 8.1 Hz, 1H, Ph-H), 7.467 (d, J = 7.2 Hz, 1H, Ph-H), 7.192 (t, J = 7.5 Hz, 1H, Ph-H), 6.830 (s, 1H, NH), 4.503-4.607 (m, 4H, $-CH_2-$), 4.194 (t, J = 7.2 Hz, 2H, $N-CH_2$), 3.043-3.125 (m, 4H, $-CH_2-$), 1.741 (m, 2H, $N-CH_2-CH_2$), 1.482 (m, 2H, $-CH_2-CH_3$), 1.305 (s, 12H, $-CH_3$), 0.975 (t, 3H, CH_3). ^{13}C NMR (75 MHz, $CDCl_3$, ppm): δ 164.176, 163.668, 153.522, 153.260, 139.087, 136.846, 132.310, 131.193, 130.244, 128.867, 128.506, 126.733, 126.522, 123.406, 123.213, 118.075, 117.455, 83.942, 63.178, 63.035, 40.217, 37.600, 36.972, 31.576, 30.208, 24.818, 22.639, 20.386, 14.106, 13.842. Mass spectrometry (ESI-MS, m/z): $[M + H]^+$ calcd for $C_{34}H_{41}BN_3O_8S_2$, 694.2429; found, 694.2428.

***Synthesis of NA-C*:** The compound NA-C was synthesized using the same procedure as in the synthesis of NA-S. NA-C was afforded as a brown solid (120 mg): Yield 45%. Melting point: 156-160 °C. ^{1}H NMR (300 MHz, DMSO-d_6, ppm): δ 10.232 (s, 1H, NH), 8.715 (d, J = 8.7 Hz, 1H, Ph-H), 8.496 (m, 2H, Ph-H), 8.192 (d, J = 8.4 Hz, 1H, Ph-H), 7.845 (t, J = 8.4 Hz, 1H, Ph-H), 4.365 (t, 1H, OH), 4.203 (t, J = 6.6 Hz, 2H, $O-CH_2-$), 4.049 (t, J = 7.5 Hz, 2H, $OH-CH_2$), 3.403 (m, 2H, CH_2), 1.599-1.726 (m, 4H, CH_2), 1.322-1.479 (m, 8H, CH_2), 0.934 (t, 3H, CH_3). ^{13}C NMR (75 MHz, DMSO-d_6, ppm): δ 163.879, 163.321, 154.579, 141.301, 132.095,

131.275, 129.721, 128.736, 126.690, 124.181, 122.609, 118.393, 117.297, 65.540, 61.088, 32.912, 30.127, 28.977, 25.740, 25.693, 20.265, 14.161. Mass spectrometry (ESI-MS, m/z): $[M + H]^+$ calcd for $C_{23}H_{29}N_2O_5$, 413.2076; found, 413.2071.

***Synthesis of NA-C-BAP*:** The compound NA-C-BAP was synthesized using the same procedure as in the synthesis of NA-S-BAP. NA-C-BAP was afforded as a brown solid (180 mg): Yield 62%. Melting point: 138-140 °C. ^1H NMR (300 MHz, $CDCl_3$, ppm): δ 8.612 (t, 2H, Ph-H & NH), 8.369 (d, J = 8.4 Hz, 1H, Ph-H), 8.245 (d, J = 7.8 Hz, 1H, Ph-H), 7.648 (s, 3H, Ph-H), 7.510 (d, J = 7.2 Hz, 1H, Ph-H), 7.311 (d, J = 7.5 Hz, 1H, Ph-H), 6.715 (s, 1H, NH), 4.293 (t, J = 6.9 Hz, 2H, CH_2), 4.189 (m, 4H, CH_2), 1.683-1.784 (m, 6H, CH_2), 1.494 (m, 6H, CH_2), 1.335 (s, 12H, $-CH_3$), 0.989 (t, 3H, CH_3). ^{13}C NMR (75 MHz, DMSO-d_6, ppm): δ 163.916, 163.360, 154.600, 154.114, 141.300, 139.232, 132.127, 131.312, 129.773, 128.708, 126.747, 124.277, 122.663, 121.754, 118.547, 117.391, 84.079, 65.476, 64.527, 30.140, 28.936, 28.858, 25.526, 25.134, 20.264, 14.171. Mass spectrometry (ESI-MS, m/z): $[M + H]^+$ calcd for $C_{36}H_{45}BN_3O_8$, 658.3300; found, 658.3301.

***Synthesis of CPT-S*:** A mixture of triphosgene (40 mg, 0.13 mmol), DMAP (122 mg, 1 mmol) and CPT (100 mg, 0.29 mmol) in 10 mL of anhydrous chloroform was stirred under argon protection for 0.5 h at room temperature. Thereafter, a solution of 2,2'-dithiodiethanol (77 mg, 0.45 mmol) in anhydrous chloroform was added. The resulting mixture was stirred overnight at room temperature. After the removal of the solvent under reduced pressure, the crude product was purified by flash silica gel chromatography using dichloromethane/methanol (v/v, 1:0 to 40:1) as the eluent to afford CPT-S as a white solid (90 mg): Yield 58.8%. Melting point: 168-170 °C. ^1H NMR (300 MHz, $CDCl_3$, ppm): δ 8.448 (d, J = 9.0 Hz, 1H, Ph-H), 8.244 (d, J = 8.4 Hz, 1H, Ph-H), 7.908 (m, 1H, Ph-H), 7.857 (m, 1H, Ph-H), 7.450-7.740 (m, 2H, Ph-H), 5.753 (m, 1H), 5.300-5.460 (m, 3H), 4.412 (m, 2H), 3.942 (m, 2H), 2.800-3.020 (m, 4H), 2.000-2.400 (m, 1H), 1.902 (m, 1H), 1.041 (m, 3H). ^{13}C NMR (75 MHz, $CDCl_3$, ppm): δ 167.396, 157.286, 153.500, 148.675, 146.259, 145.668, 131.494, 130.956, 129.341, 128.566, 128.234, 120.319, 96.356, 78.051, 67.105, 66.454, 60.477, 60.383, 50.025, 41.273, 41.200, 37.157, 31.901,

29.693, 7.622. Mass spectrometry (ESI-MS, m/z): $[M + H]^+$ calcd for $C_{25}H_{25}N_2O_7S_2$, 529.1103; found, 529.1081.

***Synthesis of CPT-S-BAP*:** The compound CPT-S-BAP was synthesized using the same procedure as in the synthesis of NA-S-BAP. CPT-S-BAP was afforded as a white solid (20 mg): Yield 65%. Melting point: 240-245 °C. ^{1}H NMR (300 MHz, DMSO-d_6, ppm): δ 9.620 (s, 1H, NH), 8.681 (s, 1H, Ph-H), 8.167 (q, 2H, Ph-H), 7.857 (t, 2H, Ph-H), 7.711 (t, J = 7.2 Hz, 1H, Ph-H), 7.511 (d, J = 6.9 Hz, 1H, Ph-H), 7.297 (t, 2H, Ph-H), 7.104 (s, 1H, Ph-H), 5.531 (s, 2H), 5.323 (s, 2H), 4.359 (t, J = 6.0 Hz, 2H, CH_2), 4.262 (t, J = 6.0 Hz, 2H, CH_2), 3.026 (m, 4H), 2.179 (q, 2H), 1.292 (s, 12H, CH_3), 0.925 (t, 3H, CH_3). ^{13}C NMR (75 MHz, DMSO-d_6, ppm): δ 167.503, 156.964, 153.618, 153.267, 152.696, 148.365, 146.739, 145.191, 139.012, 132.071, 130.262, 129.452, 129.004, 128.488, 128.201, 124.556, 119.652, 94.837, 84.107, 78.371, 66.928, 66.745, 62.369, 50.814, 37.240, 36.720, 30.786, 25.140, 8.008. Mass spectrometry (ESI-MS, m/z): $[M + H]^+$ calcd for $C_{38}H_{41}BN_3O_{10}S_2$, 774.2326; found, 774.2315.

Preparation of Au-Gal-BA

Spherical citrate-capped gold nanoparticles were first synthesized *via in-situ* reduction of Au(III) to colloidal gold in accordance to established synthetic procedures.[S2] 99 mL of $HAuCl_4 \cdot 4H_2O$ (0.50 mM) was refluxed in a round-bottom flask. Thereafter, 1 mL of sodium citrate solution (0.17 mM) was added dropwise at a constant rate of addition. The reaction mixture was further refluxed for 30 mins, then cooled to room temperature and stored for further use. Thiol-modified polyethylene glycol-linked β-galactoside (β-gal-PEG-SH) was synthesized as reported in the literature.[S3] In order to form β-galactose-coated Au nanoparticles, 40 μL of citrate-stabilised Au nanoparticles (0.5 mM) was added slowly to 20 μL of β-gal-PEG-SH (100 mM) and stirred for 72 h. The sample was centrifuged and washed twice with water. Na-S-BA (1.23 mg) was dissolved in 40 μL DMSO and added to the Au-Gal nanoparticles, and the sample was incubated for 12 h. The complex was centrifuged and washed with DMSO before resuspension in 20 μL DMSO to form Au-Gal-BA. The loading amount was calculated from the decrease in the concentration of NA-S-BA (or CPT-S-BA) in the diluted supernatant, as

determined by UV-Vis absorption spectra at 373 nm (or 364 nm), before and after the incubation with gold nanoparticles. Accordingly, the loading amount of NA-S-BA and CPT-S-BA was calculated to be approximately 2.4 wt % and 4.1 wt %, respectively.

Cell culture

HepG2, HeLa and Human Dermal Fibroblast (HDF) cells were cultured in Dulbecco's Modified Eagle Medium (DMEM) (Gibco) supplemented with 10% fetal bovine serum (FBS), 10,000 U/mL penicillin, 10,000 μg/mL streptomycin, 2 mM L-glutamine and 0.1 mM non-essential amino acids (NEAA). NIH3T3 cells were cultured in DMEM supplemented with 10% bovine serum, 10,000 U/mL penicillin and 10,000 μg/mL streptomycin. All cells were maintained in a 5% CO_2 humidified chamber at 37 °C. Cells were sub-cultured regularly with 0.025% trypsin-EDTA.

Flow cytometry

HepG2 cells were seeded in 6-well plates at a density of 4×10^5 cells per well one day prior to experiment. The cells were washed once with PBS and DMEM was replenished. The Au-Gal-BA complexes were added at increasing concentrations to each well and the cells were allowed to incubate for 2 h. Thereafter, the media was aspirated and the cells were washed once with PBS. For flow cytometry experiments, the cells were detached with 250 μL of 0.05% trypsin-EDTA, and the reaction was quenched with the addition of 750 μL of 10% FBS/ PBS. The cell suspension was transferred to pressure tubes and the fluorescence was analysed on the BD LSRFortessa X-20 flow cytometer (BD Biosciences). The violet laser was used for the excitation of the fluorophore and fluorescence was recorded on the AmCyan channel (BD 525/50 filter), with a total of 10,000 events recorded for each sample.

Cell viability

Cells were seeded in 96-well plates at a density of 1×10^4 cells per well in triplicate one day

prior to experiment. After the addition of Au-Gal-BA complexes, the cells were incubated for 2 h. The media was then aspirated and 100 µL (-)-phenol red DMEM and 10 µL of MTT 3-(4,5-dimethylthiazol-2-yl)-2,5-diphenyltetrazolium bromide (Molecular Probes) were added to each well. The cells were further incubated for another 4 h at 37 °C. Thereafter, 85 µL of the media was removed and 50 µL DMSO was added to solubilize the purple formazan crystals. Absorbance was quantified on the Tecan Infinite 200 PRO microplate spectrometer at an absorbance wavelength of 540 nm. Cell viability was expressed as a percentage against the control well of non-treated samples.

Confocal microscopy

Cells were seeded in glass-based confocal dishes (Nunc) one day prior to experiment. 10 µM Au-Gal-BA complex was added and the cells were incubated for 2 h. The cells were washed twice with PBS and fixed with 4% paraformaldehyde. Thereafter, the cells were stained with Alexa Fluor 633 phallodin (10 µL per sample) and incubated at 37 °C for 1 h. Cell images were acquired using an excitation wavelength of 488 nm and 633 nm, and emission filter in the range of 501- 602 nm and 638- 747 nm for the imaging of Au-Gal-BA and Alexa Fluor 633 phalloidin respectively. For intracellular tracking, the cells were placed in media and incubated with LysoTracker Red DND-99 (0.1 µM), MitoTracker Deep Red FM (200 nM) or ER-Tracker Red (1 µM) (Molecular Probes) in accordance to manufacturer's instructions. Fluorescence imaging was conducted on a confocal laser scanning microscope (Zeiss LSM 710).

Preparation of Au-Gal-BA(CPT)

30 µL of citrate-stabilised Au nanoparticles (0.5 mM) added slowly to 15 µL of β-gal-PEG-SH (100 mM) and stirred for 72 h. The sample was centrifuged and washed twice with water. CPT-S-BA (1.04 mg) was dissolved in 30 µL DMSO and added to the Au-Gal nanoparticles, and the sample was incubated for 12 h. The complex was centrifuged and washed with DMSO before resuspension in 15 µL DMSO to form Au-Gal-BA(CPT).

Cytotoxicity of Au-Gal-BA(CPT) pro-drug

Cells were seeded in 96-well plates at a density of 1 × 10^4 cells per well in triplicate one day prior to experiment. Au-Gal-BA(CPT) was added at increasing concentrations and the cells were allowed to incubate at 37 °C for 72 h. After the cells were washed with PBS, DMEM (100 µL) was replenished and 10 µL of WST-1 cell proliferation reagent (Roche) was added. The cells were incubated for 2 h and absorbance was quantified on the Tecan Infinite 200 PRO microplate spectrometer at a wavelength of 450 nm (reference wavelength 650 nm). Cell viability was expressed as a percentage against the control well of non-treated samples.

Annexin V/ propidium iodide (PI) assay

HepG2 cells were seeded in 6-well plates at a density of 4 × 10^5 cells per well one day prior to experiment. Au-Gal-BA(CPT) and CPT were added to each well respectively and the cells were incubated at 37 °C for 72 h. The media was aspirated and the cells were washed once with PBS, then harvested with 0.05 % trypsin-EDTA (250 µL). The reaction was quenched with the addition of 10% FBS/ PBS (750 µL) and transferred to pressure tubes. The supernatant formed after centrifugation was discarded, and the cells were resuspended in 1X annexin-binding buffer (100 µL). Annexin V Alexa Fluor 488 and PI (Invitrogen) were added in accordance to manufacturer's instructions (5 µL and 1 µL respectively), and the cells were incubated in the dark for 15 min. Thereafter, the cell suspension was further diluted in 1X annexin-binding buffer (400 µL) before analysis on the BD LSRFortessa X-20 flow cytometer (BD Biosciences). Fluorescence of Alexa Fluor 488 and PI were measured on the FITC and PE-CF594 channels (488 nm and 561 nm excitation) respectively with a total of 10,000 events recorded for each sample.

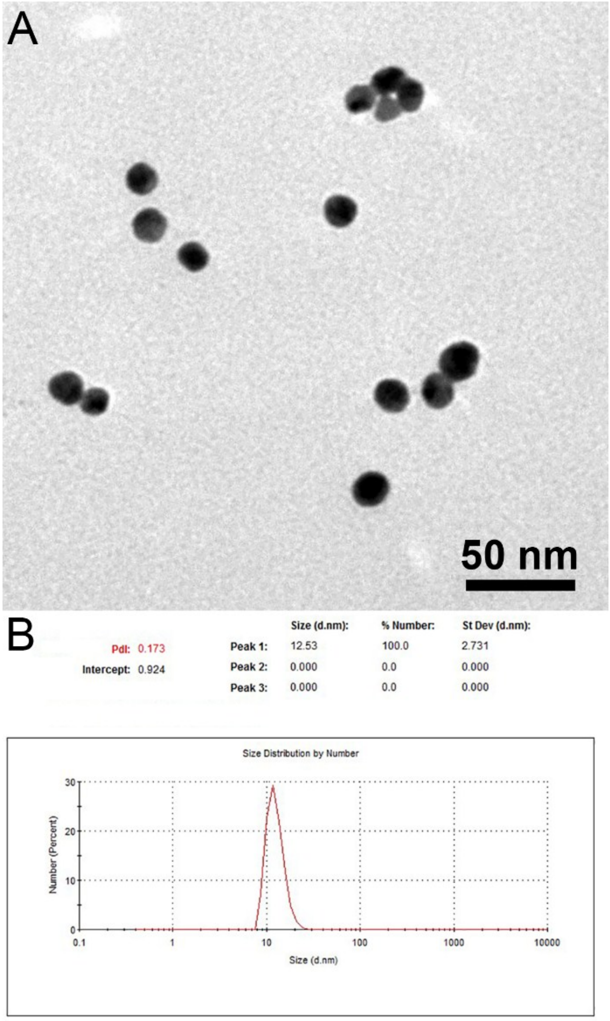

Fig. S1 (A) TEM image of gold nanoparticles and (B) hydrodynamic size distribution of the gold nanoparticles in aqueous solution measured by dynamic light scattering.

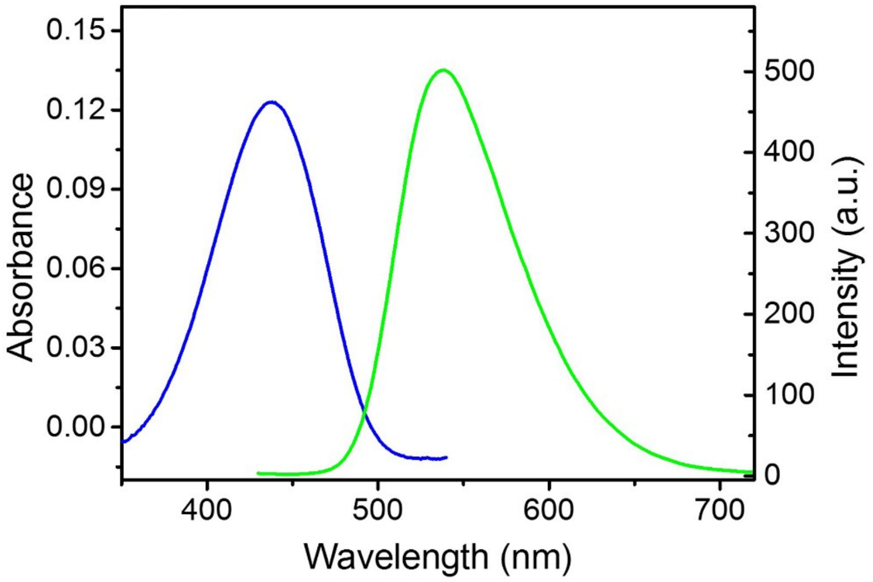

Fig. S2 Absorption and emission spectra of NA-NH_2 (10 μM) in DMSO/PBS solution (1:1, v/v, pH = 7.4, 10 mM).

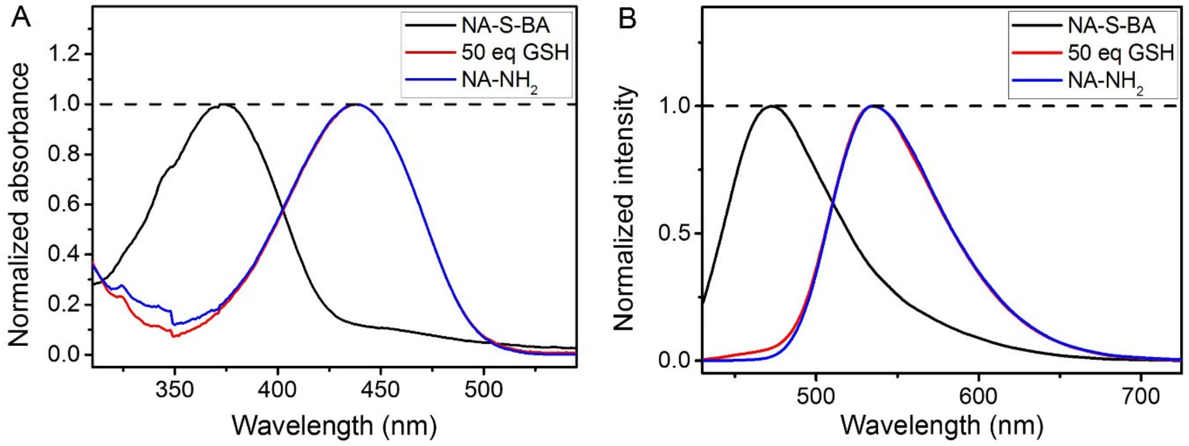

Fig. S3 Normalized absorption (A) and fluorescence (B) spectra of NA-S-BA (10 μM), NA-S-BA (10 μM, treated with 50 eq. GSH) and NA-NH_2 (10 μM) in DMSO/PBS solution (1:1, v/v, pH = 7.4, 10 mM). Spectrum of NA-S-BA with GSH was recorded 1 h after exposure at 37 °C, λ_{ex} = 405 nm.

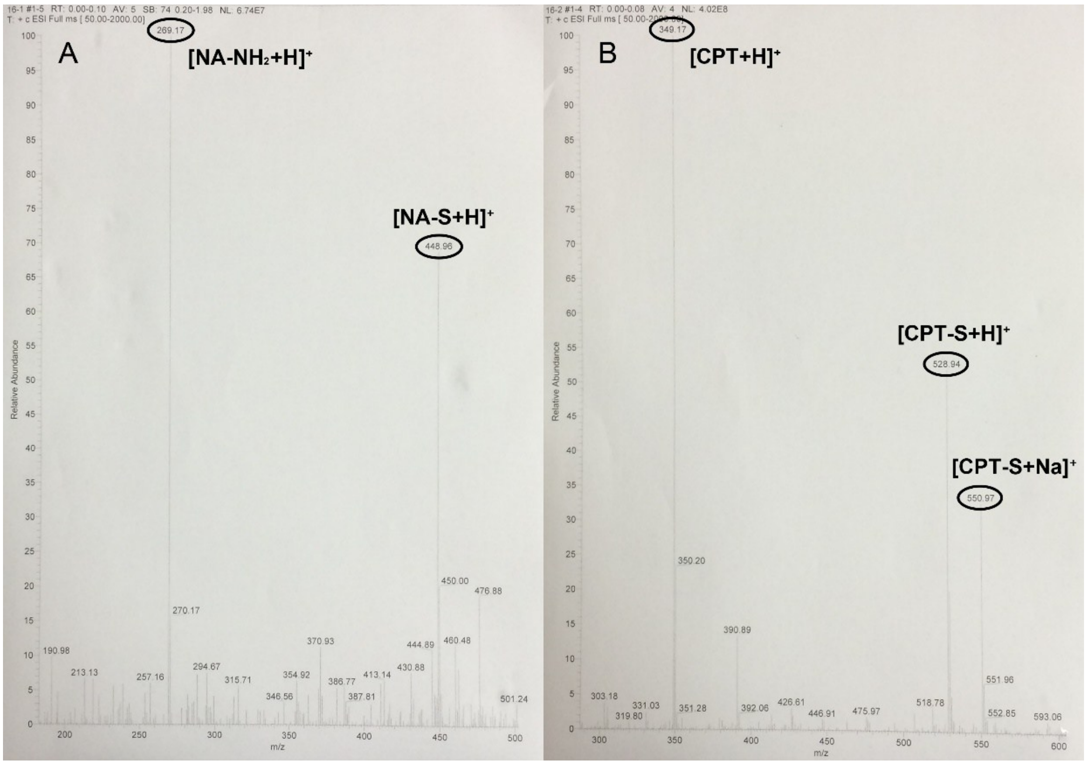

Fig. S4 MS spectrum of the products from the reaction of NA-S-BA (A) and CPT-S-BA (B) (10 μM, DMSO/PBS, 1:1, v/v, pH 7.4, 10 mM) with 20 eq. of GSH. Spectrum was obtained 1 h after exposure at 37 °C.

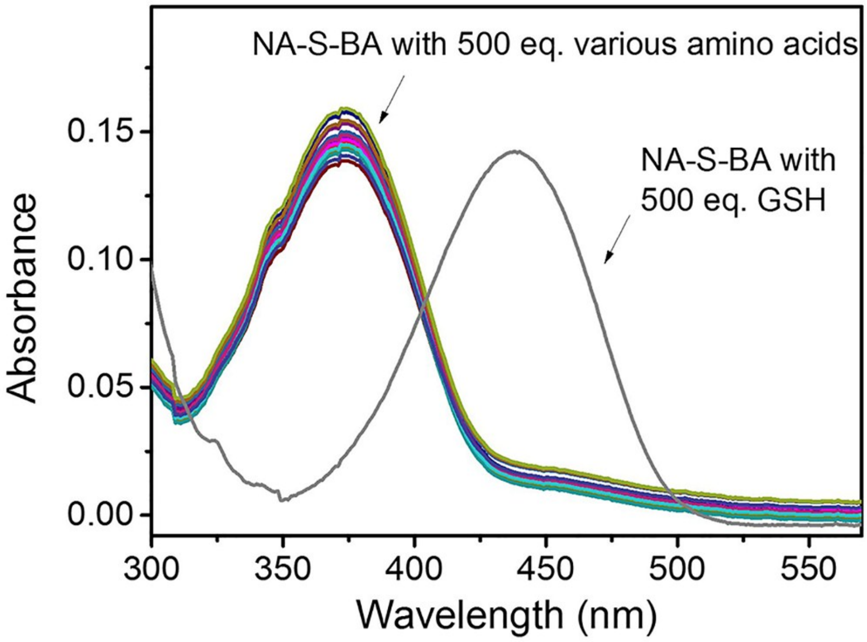

Fig. S5 Absorption response of NA-S-BA (10 μM) upon addition of various amino acids including Ala, Leu, Ile, Val, Pro, Phe, Met, Trp, Gly, Ser, Gln, Thr, Asn, Tyr, Asp, Glu, Lys,

Arg, His (5.0 mM). Each spectrum was recorded after exposure to GSH for 1 h at 37 °C.

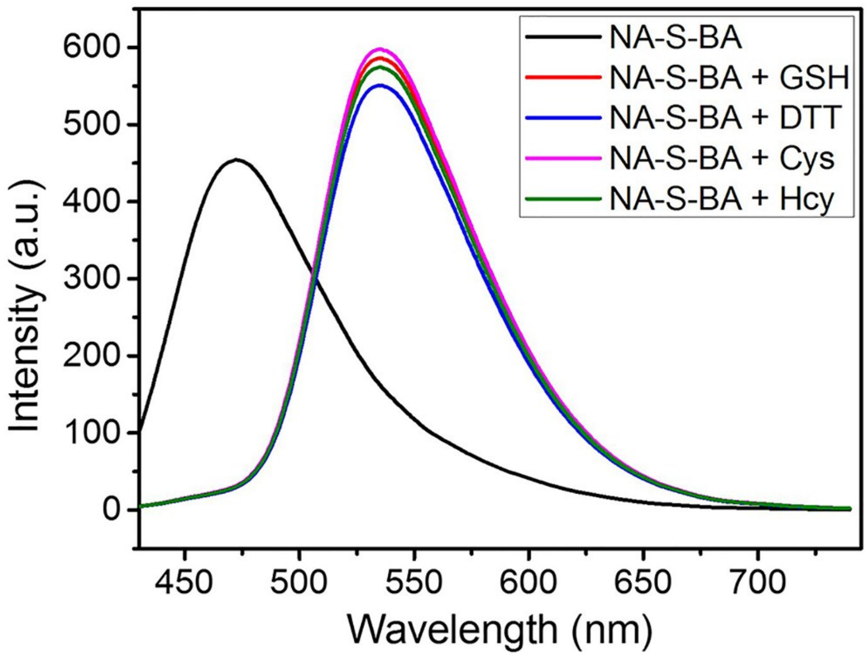

Fig. S6 Fluorescence response of NA-S-BA (10 μM) upon addition of thiol-containing compounds including GSH, DTT, Cys and Hcy (5.0 mM). Each spectrum was recorded after exposure to GSH for 1 h at 37 °C, λ_{ex} = 405 nm.

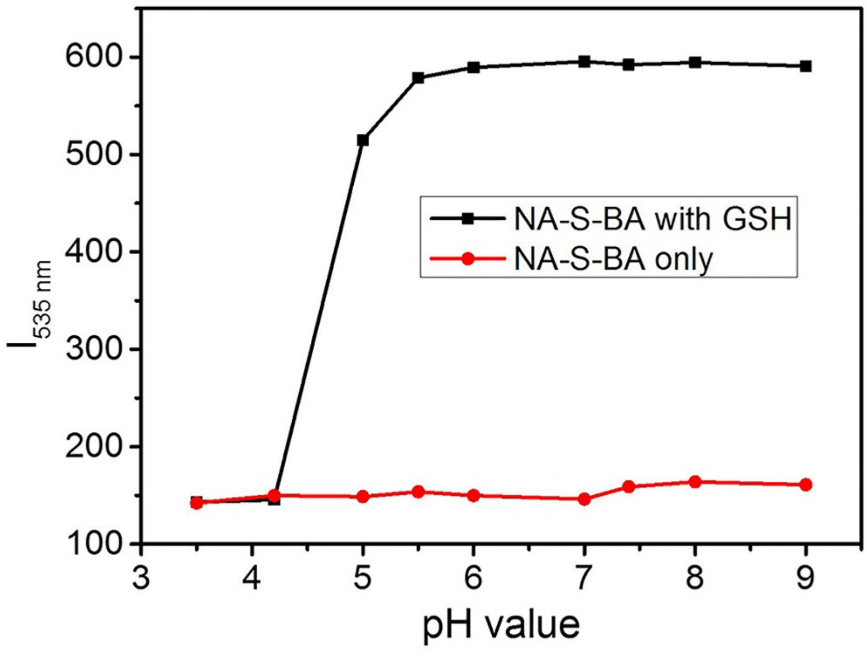

Fig. S7 Fluorescence intensity at 535 nm of NA-S-BA (10 μM) as a function of pH value in the absence and presence of GSH (5.0 mM) in DMSO/PBS solution (1:1, v/v, pH = 7.4, 10 mM).

Each point was recorded after exposure to GSH for 1 h at 37 °C, λ_{ex} = 405 nm.

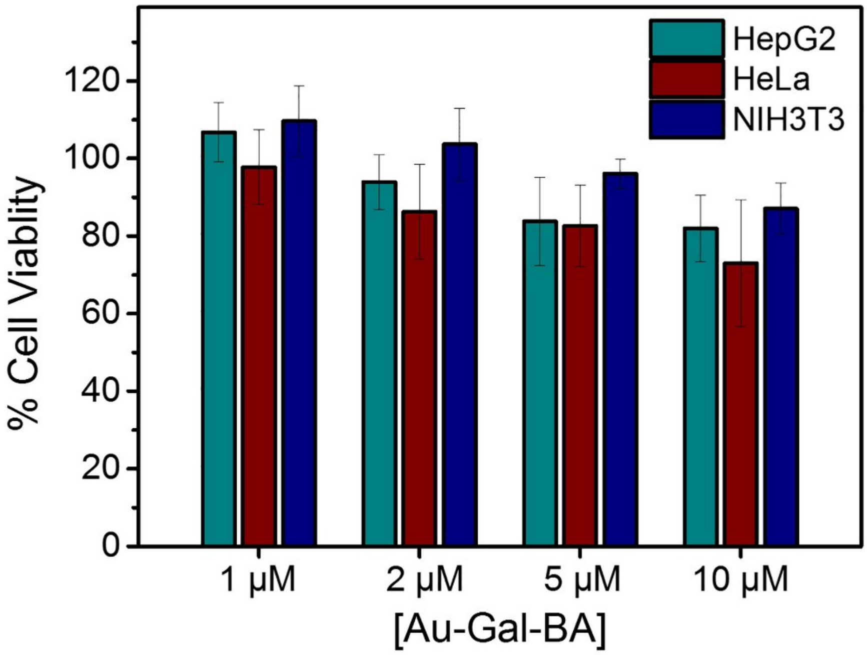

Fig. S8 Cell viability of HepG2, HeLa and NIH3T3 cells with increasing concentrations of Au-Gal-BA (from left to right, 1 μM, 2 μM, 5 μM, 10 μM). Measured using MTT assay, with absorbance quantified at 540 nm. Data is represented as mean ± SEM (n ≥ 3).

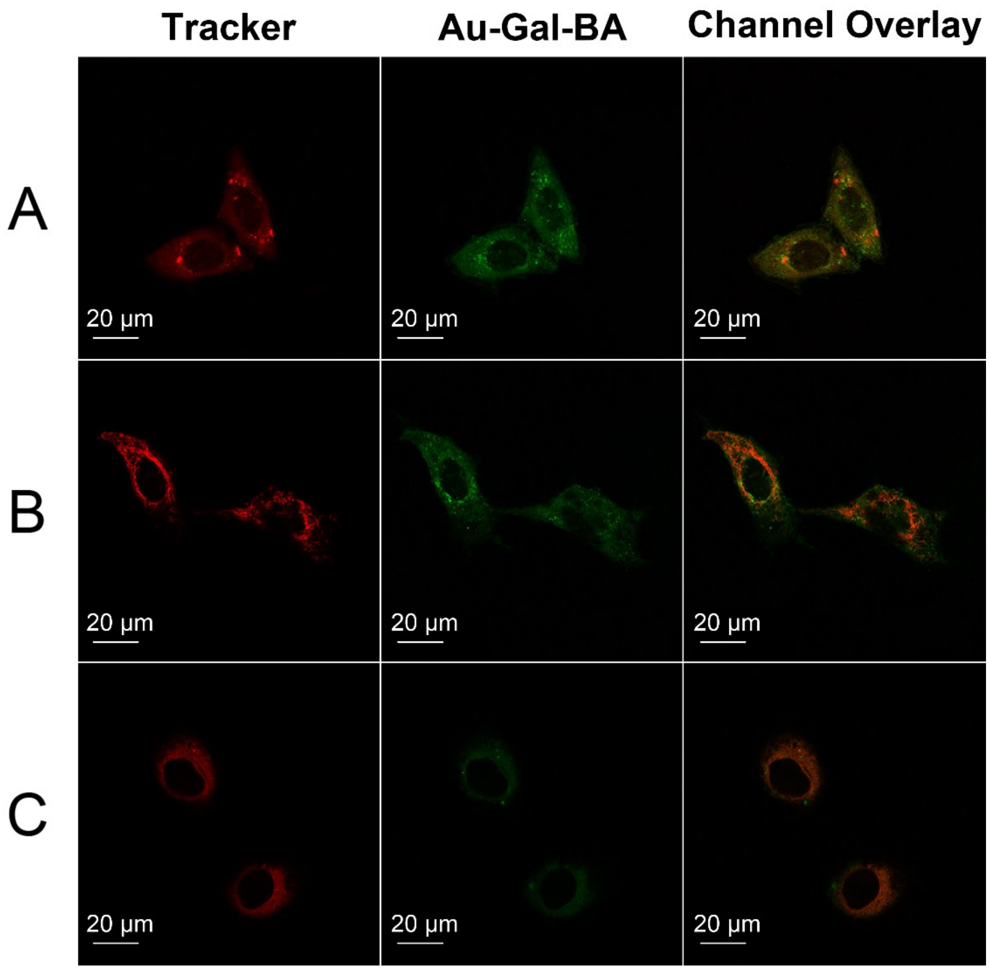

Fig. S9 Intracellular tracking of Au-Gal-BA in HepG2 cells. Fluorescence images of HepG2 cells incubated with Au-Gal-BA (labelled green) and (A) Lyso-, (B) Mito- or (C) ER-tracker; merged images.

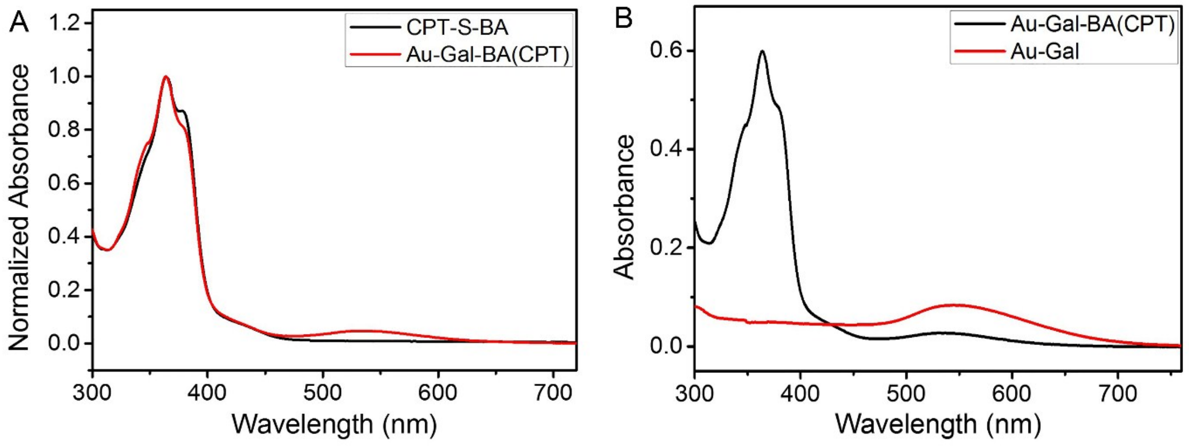

Fig. S10 The comparison of the absorption spectra of (A) CPT-S-BA with gold nanoparticles

conjugated with CPT-S-BA and (B) gold nanoparticles with and without the conjugation of CPT-S-BA.

[S1] W. Xuan, R. Pan, Y. Wei, Y. Cao, H. Li, F. Liang, K. Liu, W. Wang, *Bioconjugate Chem.* **2016**, *27*, 302.

[S2] Z. Wu, J. Liang, X. Ji, W. Yang, *Colloids Surf. A* **2011**, *392*, 220.

[S3] R. Kikkeri, B. Lepenies, A. Adibekian, P. Laurino, P. H. Seeberger, *J. Am. Chem. Soc.* **2009**, *131*, 2110.

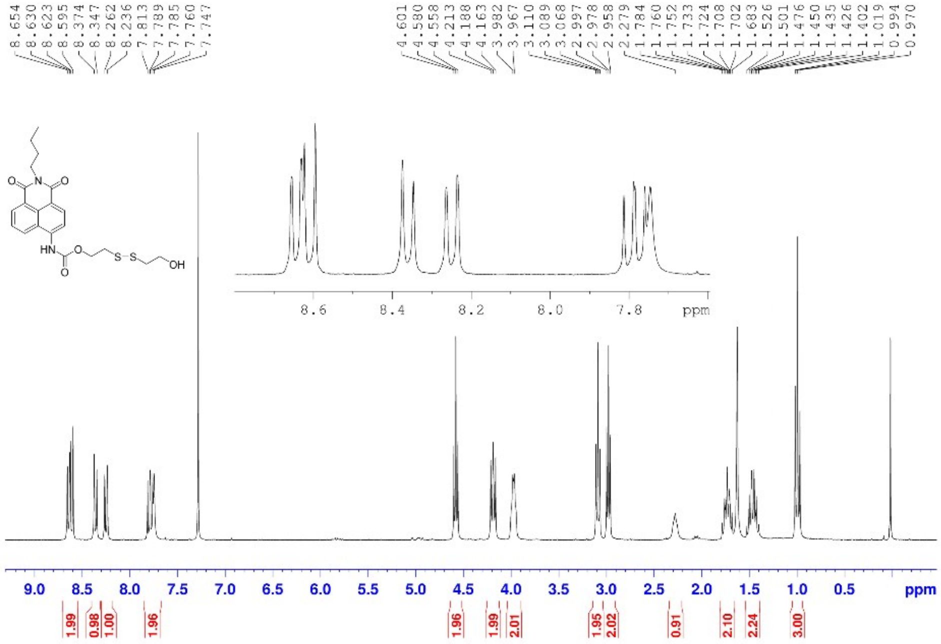

Fig. S11 ^{1}H NMR spectrum of NA-S in $CDCl_3$.

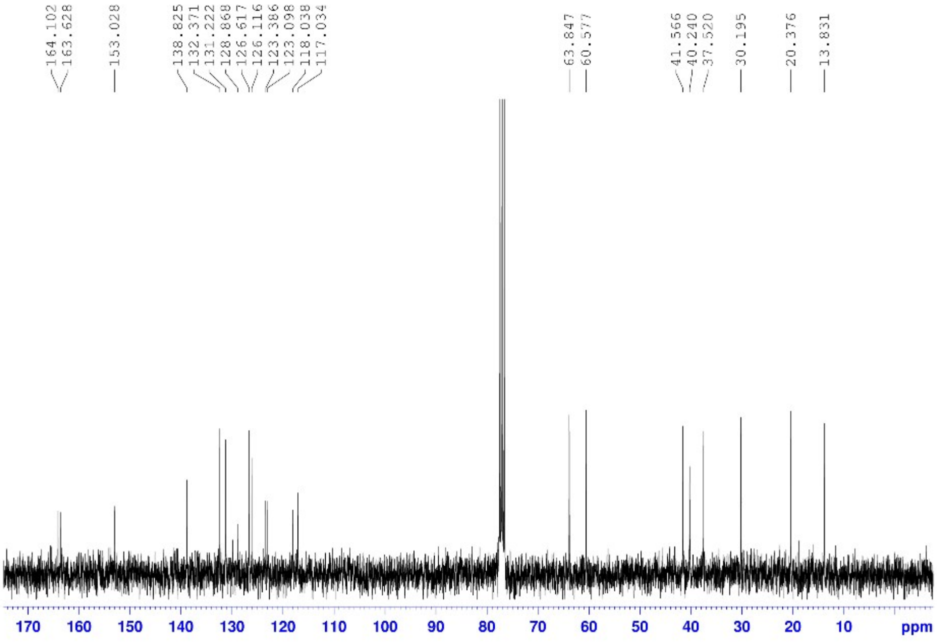

Fig. S12 ^{13}C NMR spectrum of NA-S in $CDCl_3$.

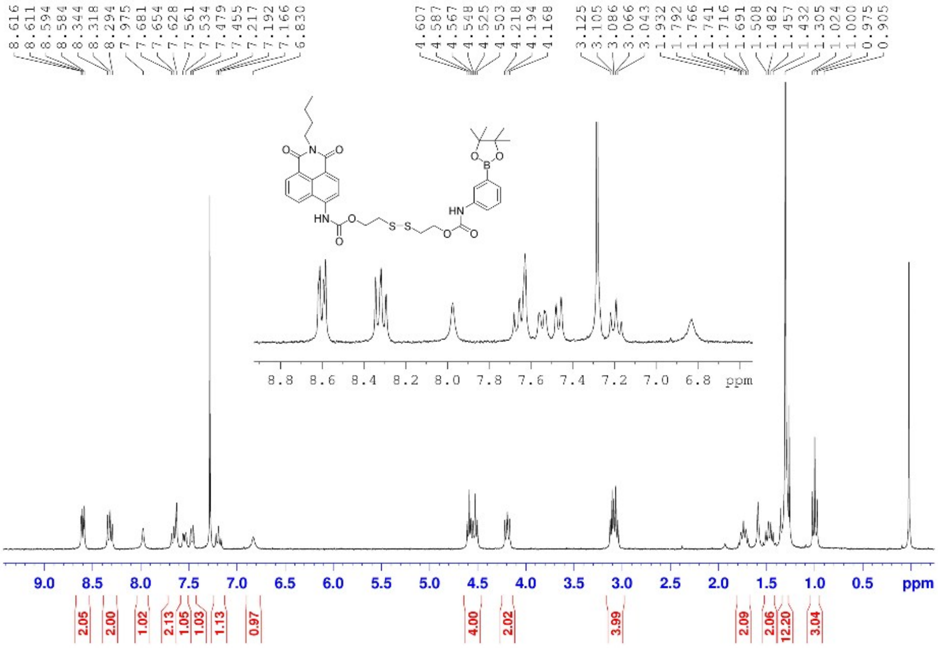

Fig. S13 ^{1}H NMR spectrum of NA-S-BAP in $CDCl_3$.

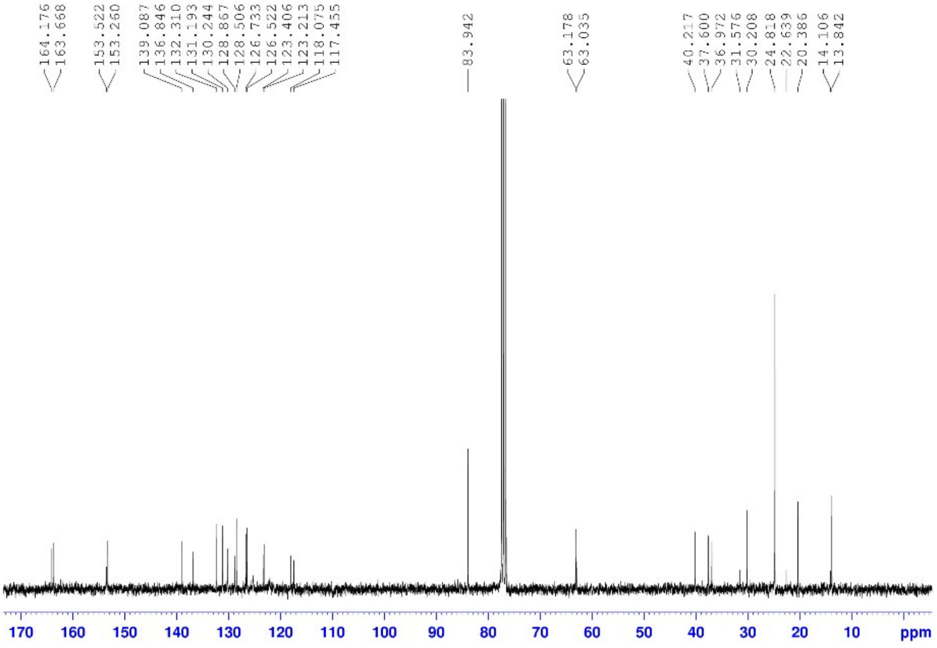

Fig. S14 ^{13}C NMR spectrum of NA-S-BAP in $CDCl_3$.

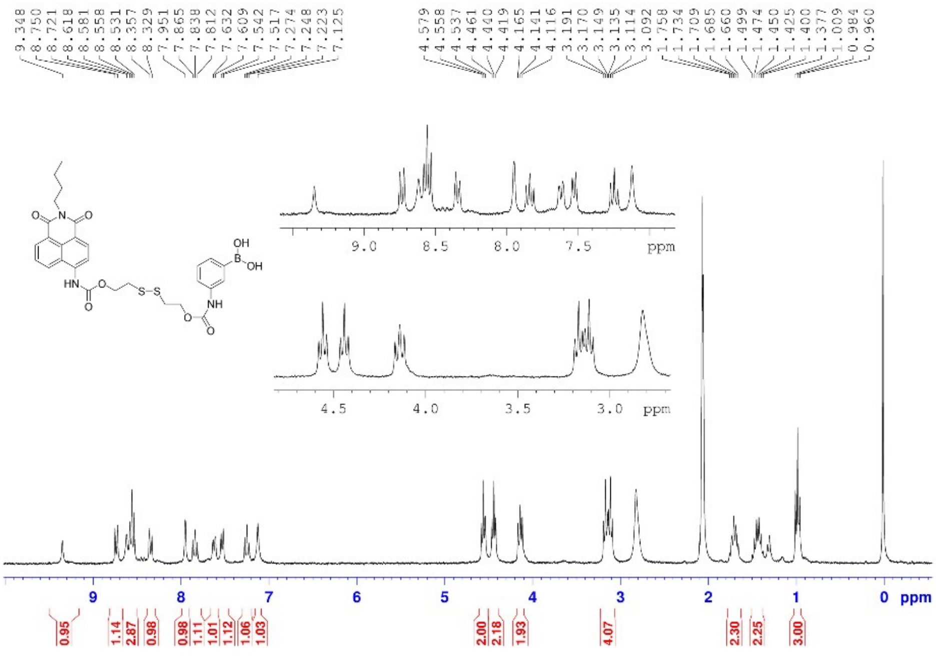

Fig. S15 ^{1}H NMR spectrum of NA-S-BA in DMSO-d_6.

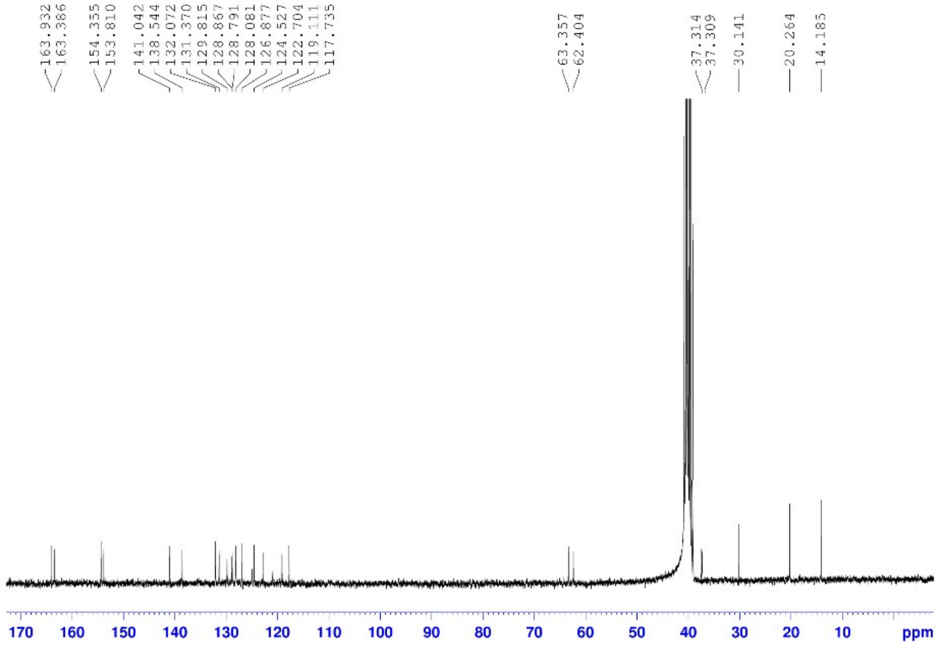

Fig. S16 ^{13}C NMR spectrum of NA-S-BA in DMSO-d_6.

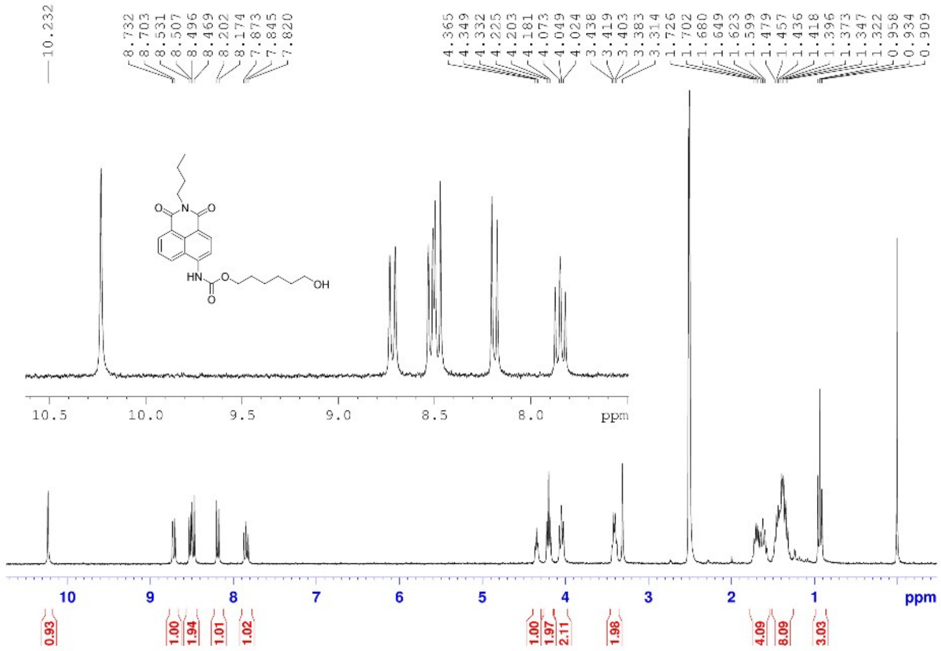

Fig. S17 ^{1}H NMR spectrum of NA-C in DMSO-d_6.

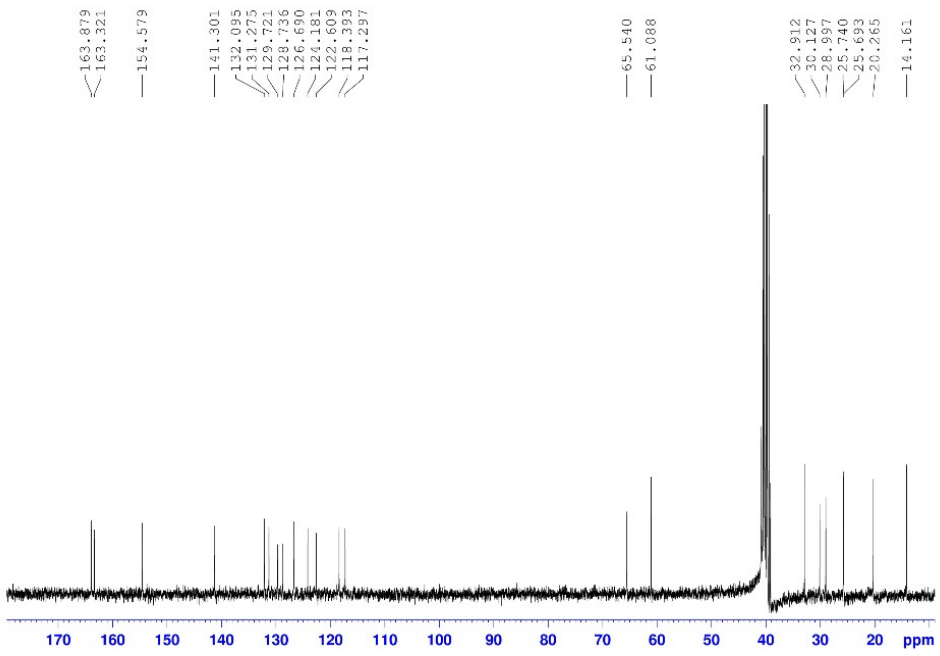

Fig. S18 ^{13}C NMR spectrum of NA-C in DMSO-d_6.

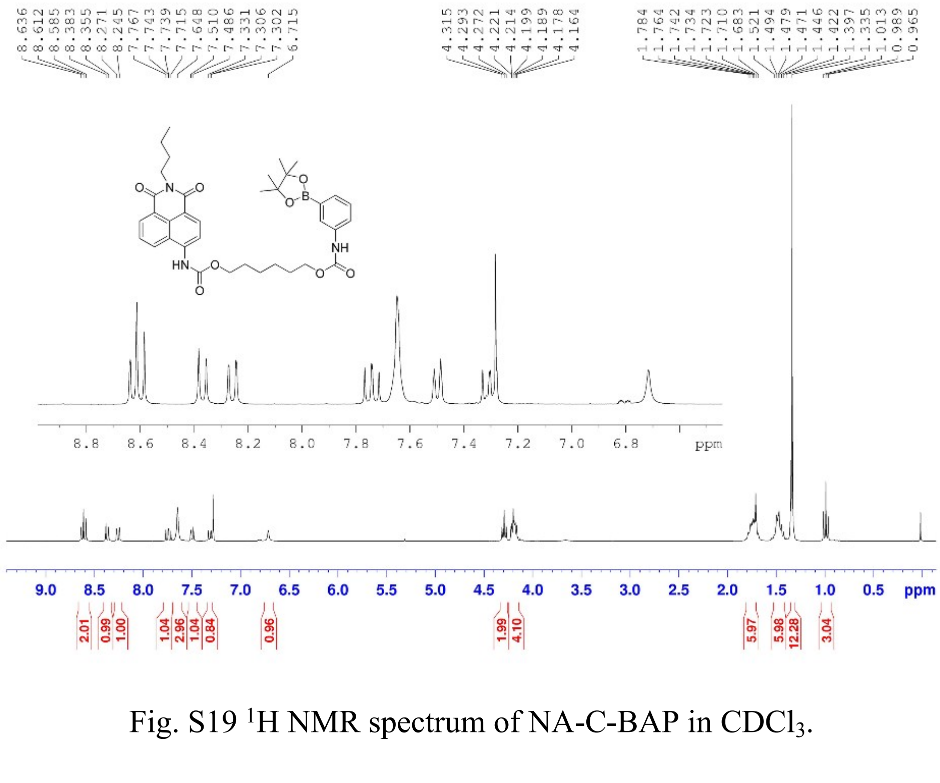

Fig. S19 ^{1}H NMR spectrum of NA-C-BAP in $CDCl_3$.

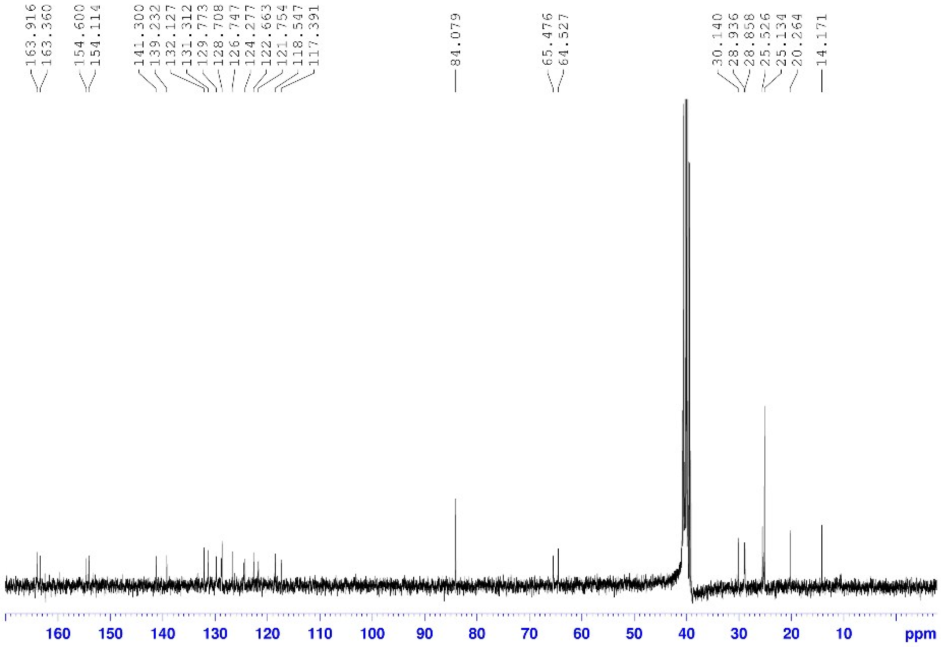

Fig. S20 ^{13}C NMR spectrum of NA-C-BAP in DMSO-d_6.

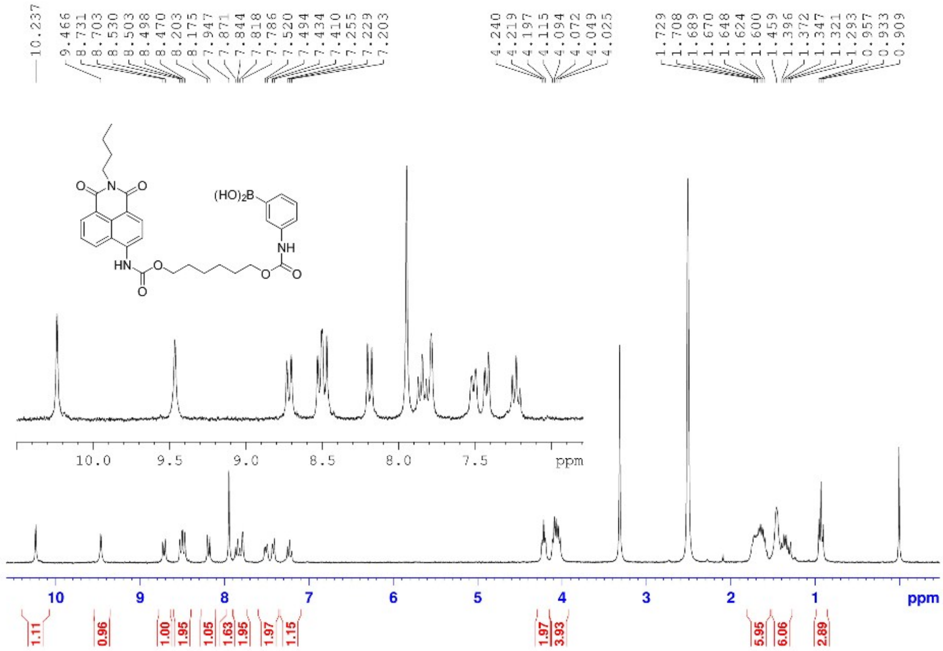

Fig. S21 ^1H NMR spectrum of NA-C-BA in DMSO-d_6.

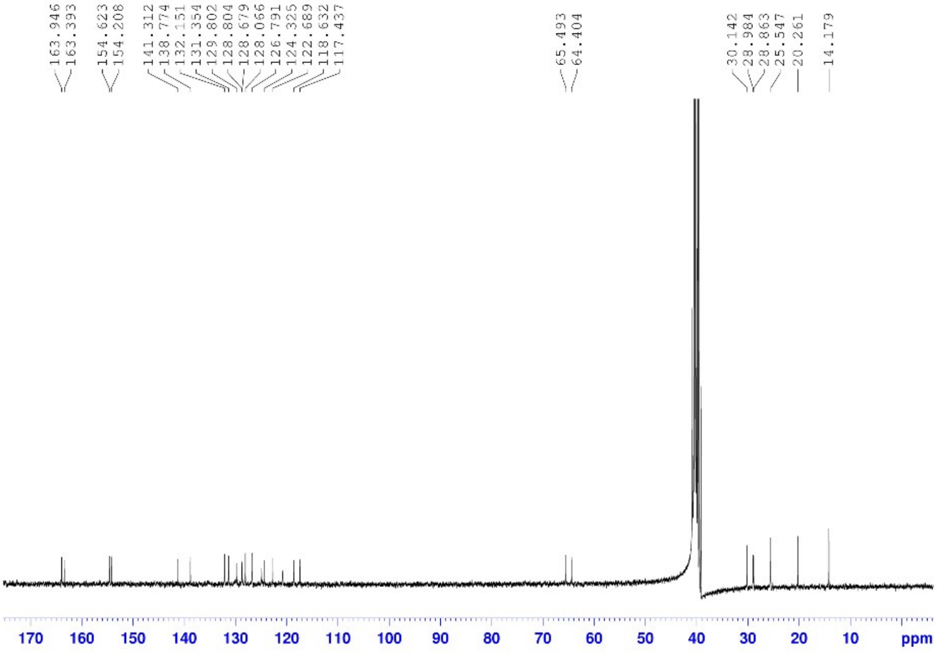

Fig. S22 ^{13}C NMR spectrum of NA-C-BA in DMSO-d_6.

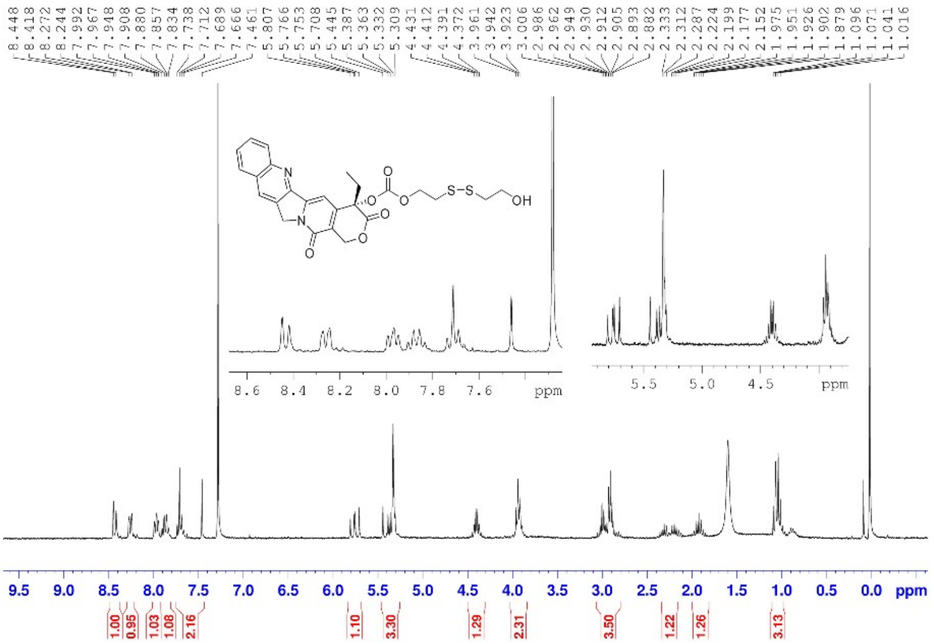

Fig. S23 ^{1}H NMR spectrum of CPT-S in $CDCl_3$.

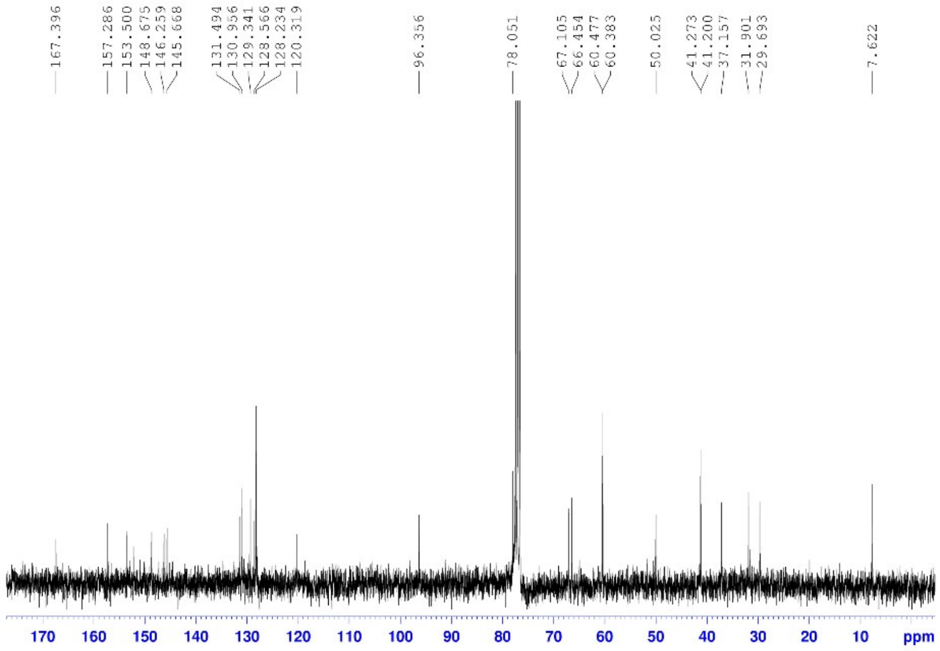

Fig. S24 ^{13}C NMR spectrum of CPT-S in $CDCl_3$.

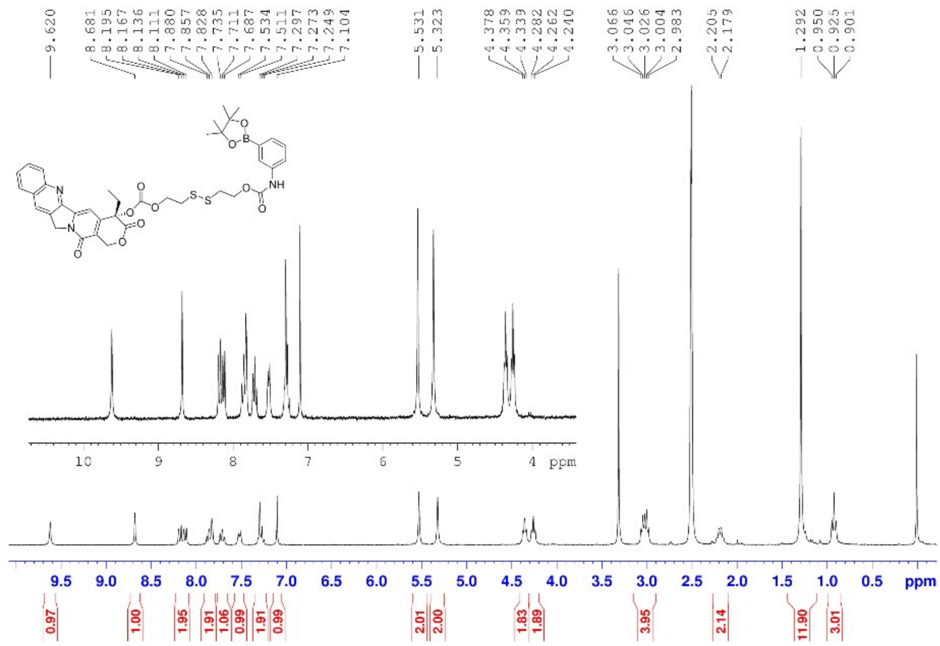

Fig. S25 ^{1}H NMR spectrum of CPT-S-BAP in DMSO-d_6.

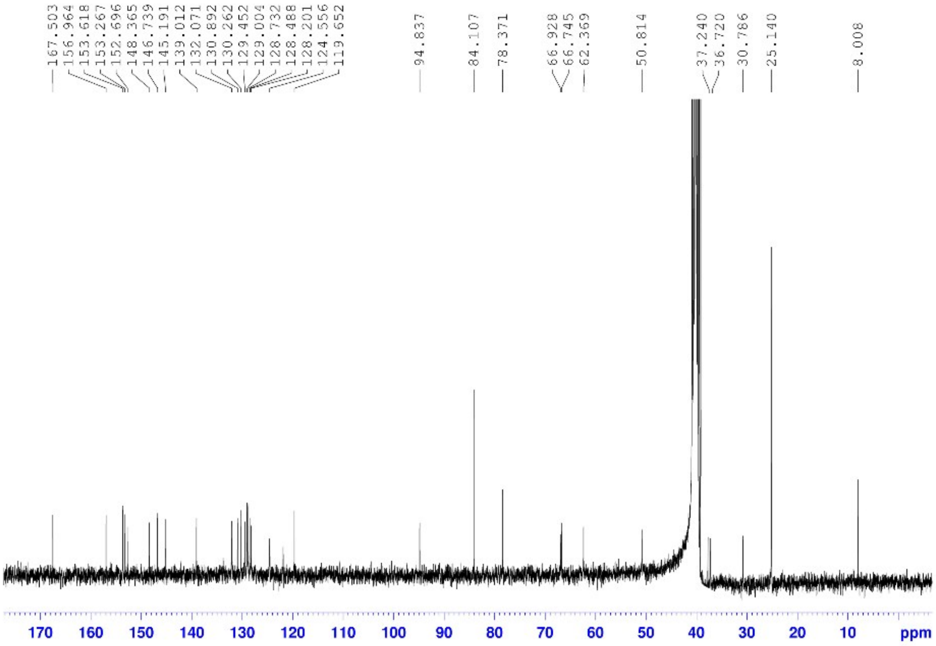

Fig. S26 ^{13}C NMR spectrum of CPT-S-BAP in DMSO-d_6.

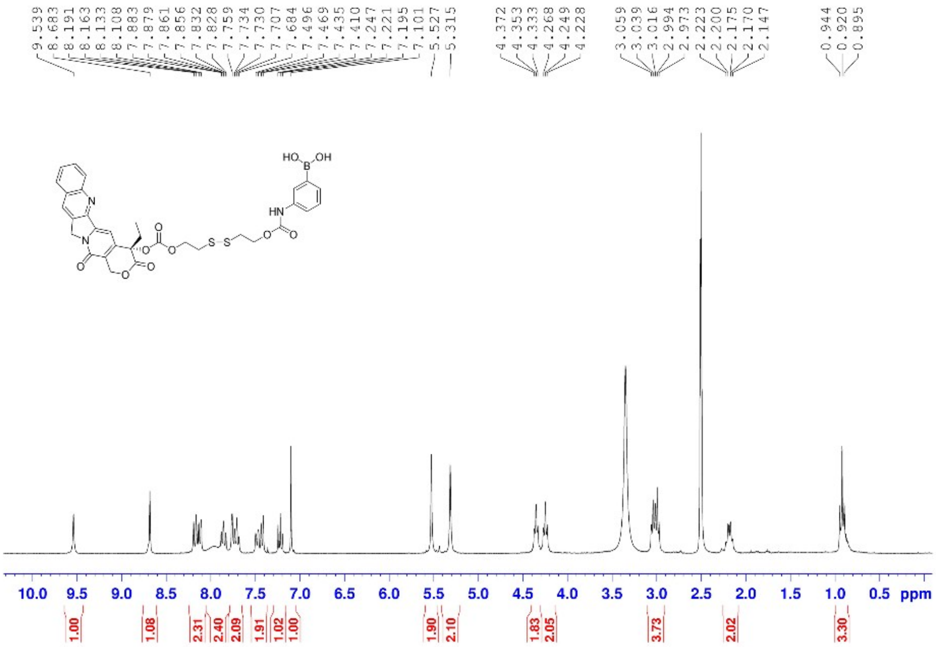

Fig. S27 ^{1}H NMR spectrum of CPT-S-BA in DMSO-d_6.

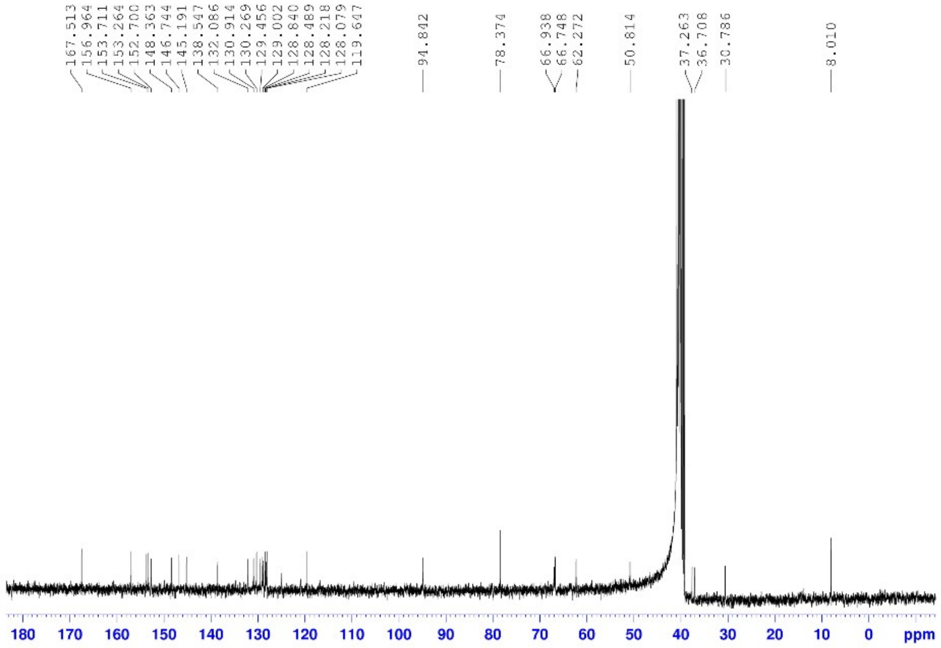

Fig. S28 ^{13}C NMR spectrum of CPT-S-BA in DMSO-d_6.
